# Supplementary material for: Novel mathematical approach to accurately quantify 3D endothelial cell morphology and vessel geometry based on fluorescently marked endothelial cell contours: Application to the dorsal aorta of wild-type and Endoglin-deficient zebrafish embryos
Source: PLoS Comput Biol. 2024 Aug 30;20(8):e1011924. doi: 10.1371/journal.pcbi.1011924 (PMC11392406; doi:10.1371/journal.pcbi.1011924)
Supplement: S1 Appendix — The text references S2–S6 Figs. (PDF) [file pcbi.1011924.s001.pdf]

S1 Appendix: Supporting methods

Novel mathematical approach to accurately quantify 3D endothelial cell morphology and vessel geometry based on fluorescently marked endothelial cell contours: Application to the dorsal aorta of wild-type and Endoglin-deficient zebrafish embryos

Daniel Seeler, Nastasja Grdseloff, Claudia Jasmin Rödel, Charlotte Kloft, Salim Abdelilah-Seyfried, Wilhelm Huisinga

Contents

1 Enrichment of endothelial cell contours **2**
1.1 Identifying points from neighboring cell contours to insert 3
1.2 Choice where to insert points from neighboring cells 3
1.3 Linear interpolation of cell contour 3

2 Determination of coordinate system **4**
2.1 Definition of coordinate system and mean shape 4
2.2 Estimation of coordinate system and mean shape 4
2.3 Initial values for coordinate system 5
2.4 Initial values for mean shape 6

3 Description of dorsal aorta cross-sections **7**
3.1 Cross-sectional shape model 7
3.2 Projection of points onto cross-section 7

4 Smoothing of vessel surface **8**

5 Equidistant points **8**
5.1 Numerical computation of equidistant points 8
5.2 Approximation of arc length of superellipse 9

6 Projection of endothelial cell contours onto vessel surface **10**

7 Triangulation within projected endothelial cell contours **11**
7.1 Collection of edges between neighboring cross-sections 11
7.2 Paths on cross-sectional shape between two edges 11
7.3 Triangulation between two paths on neighboring cross-sections 12
7.4 Refinement of triangulation 12
7.5 Extraction of cell mesh 13

8 Elongation of endothelial cells **13**
8.1 Cell surface bounding box 13
8.2 Computation of elongation 16

9 Classification of endothelial cells **16**
9.1 Plane octants 16
9.2 Plane sectors 16
9.3 Partial cell surfaces 17
9.4 Simple cell classification 17
9.5 Detailed cell classification 17

10 Criteria for the choice of tuning parameter values **17**
10.1 Preprocessing of fluorescent images 17
10.2 Vessel surface reconstruction 18

11 Unrolling of endothelial cell surfaces **19**

12 Weighted statistics **19**

1 Enrichment of endothelial cell contours

We enriched each endothelial cell contour in an iterative process (see also S1 Algorithm and Fig 2 within main text): For each cell i with $i = 1, 2, \ldots, N$, we initialized its enriched cell contour $\mathrm{EC}_{\mathrm{enri},i}$ with the corresponding manually annotated cell contour $\mathrm{EC}_{\mathrm{anno},i}$. We then iterated over all points found on other manually annotated cell contours $\mathrm{EC}_{\mathrm{anno},i'}$ with $i' \neq i$. For each point $q \in \mathrm{EC}_{\mathrm{anno},i'}$, we checked whether q was located within a cylinder around an edge (p, p^+) of the current version of the enriched cell contour $\mathrm{EC}_{\mathrm{enri},i}$. If it was, we updated $\mathrm{EC}_{\mathrm{enri},i}$ by inserting q into this contour. As in each iteration we always placed cylinders around the edges of the current version of the enriched cell contour of cell i, the final version of this enriched cell contour depended on the order of the cells i' and their points q in which they were considered for enrichment. For simplicity, we processed cells and the points on them in the order of their manual annotation. After all information from neighboring cell contours was inserted into $\mathrm{EC}_{\mathrm{enri},i}$, we further added points on edges of this contour by linear interpolation. We now explain the details of the enrichment algorithm.

S1 Algorithm: Endothelial cell contour enrichment

Input : manually annotated cell contours $\mathrm{EC}_{\mathrm{anno}}$, index i of cell to be enriched
Output : enriched cell contour $\mathrm{EC}_{\mathrm{enri},i}$ of cell i

`// Initialize enriched contour with the manual annotation`
$\mathrm{EC}_{\mathrm{enri},i} \leftarrow \mathrm{EC}_{\mathrm{anno},i}$
`// Enrichment of cell contour by neighboring cells`
for $i' = 1, 2, \ldots, N;\ i' \neq i$ **do**
  for $q \in EC_{anno,i'}$ **do**
    if *q is located in any cylinder around an edge (p, p^+) of $EC_{enri,i}$* **then**
      identify edge $(p^*, (p^*)^+)$ of $\mathrm{EC}_{\mathrm{enri},i}$ where to insert q
      `// Insert` q `into` $\mathrm{EC}_{\mathrm{enri},i}$
      either: $\mathrm{EC}_{\mathrm{enri},i} \leftarrow (\ldots, (p^*)^-, p^*, \mathbf{q}, (p^*)^+, \ldots)$
      or: $\mathrm{EC}_{\mathrm{enri},i} \leftarrow (\ldots, (p^*)^-, \mathbf{q}, p^*, (p^*)^+, \ldots)$

`// Linear interpolation of cell contour`
for $p \in EC_{enri,i}$ **do**
  linearly interpolate $n_{\mathrm{interp}}(p)$ points $q_p(j)$ on contour edge (p, p^+)
  `// Insert interpolated points` $q_p(j)$ `into` $\mathrm{EC}_{\mathrm{enri},i}$
  $\mathrm{EC}_{\mathrm{enri},i} \leftarrow (\ldots, p^-, p, q_p(1), q_p(2), \ldots, q_p(n_{\mathrm{interp}}(p)), p^+, \ldots)$

1.1 Identifying points from neighboring cell contours to insert

To decide whether to insert $q \in \mathrm{EC}_{\mathrm{anno},i'}$ into the current version of the enriched cell contour $\mathrm{EC}_{\mathrm{enri},i}$ of cell i, we first checked whether q was located in any cylinder centered around an edge (p, p^+) of points $p, p^+ \in \mathrm{EC}_{\mathrm{enri},i}$. For each edge (p, p^+), we computed the orthogonal projection $\Pi_p^{\mathrm{line}}(q)$ of q onto the line passing through p and p^+:

$$\Pi_p^{\mathrm{line}}(q) = p + \left(\frac{p^+ - p}{||p^+ - p||_2} \cdot (q - p) \right) \frac{(p^+ - p)}{||p^+ - p||_2}. \tag{1}$$

Additionally, we computed the midpoint M_p of the edge (p, p^+) as

$$M_p = p + \frac{1}{2}\left(p^+ - p\right). \tag{2}$$

Using Eq (1), (2), we defined $\mathrm{Cyl}_i(q; \Delta h)$ as the set of points $p \in \mathrm{EC}_{\mathrm{enri},i}$, such that q was located within cylinders with length $h = ||p^+ - p||_2 + 2\Delta h$ and radius $r \in (0, \infty)$ centered around edges (p, p^+):

$$\mathrm{Cyl}_i(q; \Delta h) := \Big\{ p \in \mathrm{EC}_{\mathrm{enri},i} : \left|\left|q - \Pi_p^{\mathrm{line}}(q)\right|\right|_2 < r \;\wedge \\ \left|\left|\Pi_p^{\mathrm{line}}(q) - M_p\right|\right|_2 \leq \frac{1}{2}\left|\left|p^+ - p\right|\right|_2 + \Delta h \Big\} \tag{3}$$

Here, we introduced a cylinder length extension $\Delta h > 0$ to allow the enrichment with points located near sharp bends of the cell contour, i.e., points close to the contour but not located within $\mathrm{Cyl}_i(q; \Delta h = 0)$ (see also S2C Fig).

1.2 Choice where to insert points from neighboring cells

Point q could be located in multiple cylinders centered around edges of $\mathrm{EC}_{\mathrm{enri},i}$ (see S2A and S2C Fig). To decide between which two points $p, p^+ \in \mathrm{EC}_{\mathrm{enri},i}$ to insert q, we considered two scenarios: If we identified any cylinders centered around edges of $\mathrm{EC}_{\mathrm{enri},i}$ that contained q when $\Delta h = 0$, we inserted q between the points p^* and $(p^*)^+$ if the orthogonal distance of q to the line going through $(p^*, (p^*)^+)$ was minimal (see S2A and S2B Fig):

$$p^* = \underset{p \in \mathrm{Cyl}_i(q; \Delta h = 0)}{\operatorname{argmin}} \left|\left|q - \Pi_p^{\mathrm{line}}(q)\right|\right|_2. \tag{4}$$

Alternatively, if q was located in any cylinders centered around edges of $\mathrm{EC}_{\mathrm{enri},i}$ only when $\Delta h \neq 0$, we determined the point p^* with minimal distance to q (see S2C and S2D Fig):

$$p^* = \underset{p \in \mathrm{Cyl}_i(q; \Delta h \neq 0)}{\operatorname{argmin}} ||q - p||_2. \tag{5}$$

In this scenario, if the distance of $(p^*)^+$ to q was smaller than the distance of $(p^*)^-$ to q, q became the new successor of p^*, otherwise q became the new *predecessor* of p^*.

1.3 Linear interpolation of cell contour

To homogenize the point density along the enriched cell contour, we finally added $n_{\mathrm{interp}}(p)$ equidistant points by linear interpolation on each edge (p, p^+) of the enriched cell contour $\mathrm{EC}_{\mathrm{enri},i}$:

$$q_p(j) = p + \frac{j}{n_{\mathrm{interp(p)}} + 1} \cdot \frac{p^+ - p}{||p^+ - p||_2}; \quad j = 1, 2, \ldots, n_{\mathrm{interp}}(p). \tag{6}$$

2 Determination of coordinate system

2.1 Definition of coordinate system and mean shape

For a given coordinate system with orthonormal basis $B = (b_x, b_y, b_z) \in \mathrm{O}(3)$ and origin $o \in \mathbb{R}^3$, we defined the z-axis $\hat{=}$ anterior-posterior axis as the line passing through o in direction b_z. The set of vessel cross-sectional planes then lies on the set of planes parallel to the xy-plane. We defined the y-axis $\hat{=}$ dorsal-ventral axis as the line passing through o in direction b_y and the x-axis $\hat{=}$ left-right axis as the line passing through o in direction b_x. The larger a point's x-/y-coordinate, the further right/dorsal it is located within the cross-sectional plane, respectively. The larger a point's z-coordinate, the closer it is located to the fish's posterior (tail).

Within this coordinate system, we defined the mean cross-sectional shape with parameters $\bar{\theta}$ as the cross-sectional shape that has a minimal sum of residuals to all data points projected onto the xy-plane. S3 Fig visualizes the relationship between coordinate system and mean shape.

2.2 Estimation of coordinate system and mean shape

We simultaneously estimated the optimal coordinate system with orthonormal basis $B^* = \left(b_x^*, b_y^*, b_z^*\right) \in \mathrm{O}(3)$ and origin o^* and the optimal mean shape with parameters $\bar{\theta} \in \mathbb{R}^7$ (compare Eq (26)) based on the equidistant points $\mathrm{EC}_{\mathrm{spline}}$ on EC contour splines and the manually annotated basis vectors b_x^{anno}, b_y^{anno} and b_z^{anno} of the left-right, dorsal-ventral and anterior-posterior axes. During estimation, we evaluated goodness of fit (GOF) by computing the sum of distances between the xy-coordinates of cell contour spline points $\mathrm{EC}_{\mathrm{spline}}$ and their projections onto the current estimate of the mean shape with parameters θ within the current coordinate system defined by basis B and origin o:

$$\mathrm{GOF}(o, B, \theta; \mathrm{EC}_{\mathrm{spline}}) \coloneqq \sum_{p \in \mathrm{EC}_{\mathrm{spline}}} \left|\left| \left[\Pi_{o,B}^{\mathrm{coord}}(p)\right]_{xy} - \Pi_{\theta}^{\mathrm{shape}}\left(\left[\Pi_{o,B}^{\mathrm{coord}}(p)\right]_{xy}\right)\right|\right|_2, \quad (7)$$

where

$$\Pi_{o,B}^{\mathrm{coord}}(p) = (p - o)^{\top} \cdot B^{\top} \quad (8)$$

is the transformation of point $p \in \mathbb{R}^3$ into the coordinate system defined by origin o and basis B. Finally, $\Pi_{\theta}^{\mathrm{shape}}(q)$ is the projection of point $q \in \mathbb{R}^2$ onto the cross-sectional shape with parameters θ. Section 3.2 provides details on how we approximated $\Pi_{\theta}^{\mathrm{shape}}$ for our cross-sectional shape model from Eq (23).

Our cross-sectional shape from Eq (23) enforces left-right symmetry and allows, but does not enforce, dorsal-ventral asymmetry. As additionally, anterior and posterior cannot be distinguished from cell contours, we further constrained our minimization problem: During the estimation of the coordinate system, we ensured that the current basis B preserved the biologically true orientation of the DA segment by comparison with the manually annotated basis $\left(b_x^{\mathrm{anno}}, b_y^{\mathrm{anno}}, b_z^{\mathrm{anno}}\right)$. If for example $b_x \cdot b_x^{\mathrm{anno}} < 0$, the orientation of left and right were switched, so we updated $b_x \leftarrow -b_x$ (compare Eq (10)). For our cross-sectional shape from Eq (23), the direction of the dorsal-ventral basis vector b_y (together with the origin o) defined our cross-sectional shape's symmetry axis, so we additionally checked during estimation if the orientation of the current y-axis was closer to the annotated y-axis or the annotated x-axis. In the latter case $\left|b_y \cdot b_y^{\mathrm{anno}}\right| < \left|b_x \cdot b_y^{\mathrm{anno}}\right|$ held, and we updated $b_y \leftarrow b_x$ (compare Eq (11)).

In summary, we solved this minimization problem to simultaneously determine the

optimal coordinate system and mean shape:

$$\left(o^*, B^*, \bar{\theta}\right) = \underset{(o,B,\theta)\in\mathbb{R}^3\times\mathrm{O}(3)\times\mathbb{R}^7}{\operatorname{argmin}} \quad \mathrm{GOF}(o, B, \theta; \mathrm{EC}_{\mathrm{spline}}) \tag{9}$$

$$\text{subject to} \quad b_x \cdot b_x^{\mathrm{anno}} > 0; \quad b_y^{\mathrm{anno}} \cdot b_y > 0; \quad b_z \cdot b_z^{\mathrm{anno}} > 0 \tag{10}$$

$$\left|b_y \cdot b_y^{\mathrm{anno}}\right| > \left|b_x \cdot b_y^{\mathrm{anno}}\right|. \tag{11}$$

We finally centered the optimal coordinate system by moving the origin o^* onto the data's midpoint in direction b_z^*. As a consequence:

$$\left[\Pi_{o^*,B^*}^{\mathrm{coord}}\left(o^*\right)\right]_z = \frac{1}{2}\left(\min_{p\in\mathrm{EC}_{\mathrm{spline}}}\left[\Pi_{o^*,B^*}^{\mathrm{coord}}\left(p\right)\right]_z + \max_{p\in\mathrm{EC}_{\mathrm{spline}}}\left[\Pi_{o^*,B^*}^{\mathrm{coord}}\left(p\right)\right]_z\right) \tag{12}$$

$$= 0. \tag{13}$$

2.3 Initial values for coordinate system

In most data sets, i.e., embryos at either time point, we found that it was easier to automatically compute a good initial direction vector for the anterior-posterior (z-)axis than to manually annotate b_z^{anno} with sufficient precision such that a physiologically plausible coordinate system was estimated (see also final paragraph of this subsection). In the analyzed DA segments, the length in anterior-posterior direction was larger than the diameter within a vessel cross-section, so we used the first principal component of all points on endothelial cell contours as initial basis vector b_{z0} for the z-axis:

$$b_{z0} = \underset{v\in\mathbb{R}^3;\,||v||_2=1}{\operatorname{argmax}} \sum_{p\in\mathrm{EC}_{\mathrm{spline}}} \left(p \cdot v\right)^2. \tag{14}$$

The manually annotated vector b_z^{anno} of the anterior-posterior axis was still necessary to inform the correct orientation of b_{z0} (compare Eq (10)).

To complete the initial basis, we constructed two vectors perpendicular to b_{z0}: First, we determined the initial basis vector b_{y0} for the dorsal-ventral (y-)axis by projecting the manually annotated basis vector b_y^{anno} onto the plane orthogonal to the initial z-axis and then normalizing the projected basis vector $\tilde{b}_{y0}$:

$$\tilde{b}_{y0} = b_y^{\mathrm{anno}} - \left(b_y^{\mathrm{anno}} \cdot b_{z0}\right) b_{z0}, \tag{15}$$

$$b_{y0} = \frac{\tilde{b}_{y0}}{\left|\left|\tilde{b}_{y0}\right|\right|_2}. \tag{16}$$

Next, we determined the initial basis vector b_{x0} for the left-right (x-)axis by the cross product between the other two initial basis vectors:

$$b_{x0} = \frac{b_{z0} \times b_{y0}}{||b_{z0} \times b_{y0}||_2}. \tag{17}$$

To fully describe the initial coordinate system, we computed an initial origin o_0 as the (component-wise) arithmetic mean of all points on endothelial cell contour splines:

$$o_0 = \frac{1}{n_{\mathrm{spline}}} \sum_{p\in\mathrm{EC}_{\mathrm{spline}}} p. \tag{18}$$

For each data set, we visually inspected whether the initial direction vectors, especially the initial direction b_{y0} for the dorsal-ventral axis, were sufficiently precise to identify a physiologically plausible coordinate system. For this, we projected the EC contour

data onto the cross-sectional (xy-)plane of the estimated coordinate system. Within the xy-plane of a physiologically plausible coordinate system, the vessel segment's cross-sections should closely align, i.e., the variance of the distances between the projected EC contour points and the mean shape should be low. Further, large deviations of the identified dorsal-ventral axis from its manual annotation should be supported by the estimation of a mean shape with more pronounced dorsal flattening than it would have within the initial coordinate system. We however found that in data sets with less pronounced dorsal-ventral asymmetry, e.g., with circular cross-sections, precise annotation of the direction b_y^{anno} of the dorsal-ventral axis was insufficient to ensure that the estimated dorsal-ventral axis respected the fish's anatomy. During the coordinate system estimation in these data sets, we thus tightly constrained the angle between the current dorsal-ventral direction vector b_y and the projection of b_y^{anno} onto the current cross-sectional plane.

2.4 Initial values for mean shape

Within the initial coordinate system, we initialized the mean shape as a circle around the origin o_0. We determined the circle's radius R_0 such that the orthogonal distances of the xy-coordinates of cell contour spline points to the circle were minimized. For a fixed origin o_0 and a fixed basis B_0, we obtained this optimization problem to determine R_0:

$$R_0 = \underset{R>0}{\text{argmin}} \sum_{p \in \text{EC}_{\text{spline}}} \left|\left| \left[\Pi^{\text{coord}}_{o_0,B_0}(p)\right]_{xy} - \Pi^{\text{circle}}_R \left(\left[\Pi^{\text{coord}}_{o_0,B_0}(p)\right]_{xy} \right) \right|\right|_2, \tag{19}$$

where

$$\Pi^{\text{circle}}_R(q) = R \cdot \frac{q}{||q||_2} \tag{20}$$

is the orthogonal projection of $q \in \mathbb{R}^2$ onto the circle with center $(0,0)^\top$ and radius $R > 0$ and the coordinate transformation $\Pi^{\text{coord}}_{o_0,B_0}(p)$ is computed with Eq (8). Expressing this circle as a member of our cross-sectional shape model from Eq (23), we obtained the initial parameters $\bar{\theta}_0$ for our mean shape:

$$\bar{\theta}_0 = \left(\bar{m}_{x0} = 0,\ \bar{m}_{y0} = 0,\ \bar{a}_0 = R_0,\ \bar{b}_0 = R_0,\ \bar{c}_0 = R_0,\ \bar{\alpha}_0 = 1,\ \bar{\beta}_0 = 1 \right). \tag{21}$$

During the determination of the coordinate system, we set the shape's midpoint $(\bar{m}_x, \bar{m}_y)$ to the current coordinate system's origin within the cross-sectional plane. The remaining five parameters $\bar{a}, \bar{b}, \bar{c}, \bar{\alpha}, \bar{\beta}$ of the superelliptic cross-sectional shape were free to change as long as they fulfilled the constraints in Eq (24) and Eq (25).

3 Description of dorsal aorta cross-sections

3.1 Cross-sectional shape model

To account for the DA's dorsal-ventral asymmetry, we developed a novel cross-sectional shape model $\varphi\colon [0, 2\pi] \to \mathbb{R}^2$ consisting of two (half) superellipses:

$$\varphi(u) = \left([\varphi(u)]_x\, , [\varphi(u)]_y\right)^\top \tag{22}$$

$$:= \begin{cases} \begin{pmatrix} m_\mathrm{x} + c \cdot \cos^\alpha (u) \\ m_\mathrm{y} + a \cdot \sin^\alpha (u) \end{pmatrix}; & 0 \le u \le \pi/2 \\ \begin{pmatrix} m_\mathrm{x} - c \cdot \cos^\alpha (\pi - u) \\ m_\mathrm{y} + a \cdot \sin^\alpha (\pi - u) \end{pmatrix}; & \pi/2 < u \le \pi \\ \begin{pmatrix} m_\mathrm{x} - c \cdot \cos^\beta (u - \pi) \\ m_\mathrm{y} - b \cdot \sin^\beta (u - \pi) \end{pmatrix}; & \pi < u \le 3\pi/2 \\ \begin{pmatrix} m_\mathrm{x} + c \cdot \cos^\beta (2\pi - u) \\ m_\mathrm{y} - b \cdot \sin^\beta (2\pi - u) \end{pmatrix}; & 3\pi/2 < u \le 2\pi \end{cases} \tag{23}$$

where the dorsal (half) superellipse has parameter values $0 \le u \le \pi$ and the ventral (half) superellipse has parameter values $\pi \le u \le 2\pi$. The cross-sectional parameters are the semi-axis lengths $a, b, c > 0$, the shape's midpoint $(m_x, m_y) \in \mathbb{R}^2$ and the exponents $\alpha, \beta \in [0.2, 1]$ (see also Fig 3B within main text). If $\alpha = 1$ or $\beta = 1$, the respective (half) superellipse reduces to an ellipse. The more $\alpha < 1$ or $\beta < 1$ are decreased, the closer their respective (half) superellipse approximates a rectangle. We empirically chose 0.2 as the lower bound for α and β to avoid unrealistically flat superellipses. To ensure that any flattening of the cross-sectional shape would predominantly occur in the dorsal superellipse, we constrained the parameters by

$$\alpha \le \beta, \tag{24}$$

$$\frac{1}{5} b \le a \le b. \tag{25}$$

Here, we chose the factor 1/5 empirically to avoid small values of a. Finally, we defined the vector θ of shape parameters as

$$\theta := (m_\mathrm{x}, m_\mathrm{y}, a, b, c, \alpha, \beta) \in \mathbb{R}^7. \tag{26}$$

Throughout the article, we refer to shape $\varphi(u)$ with parameters θ as $\varphi(u; \theta)$.

3.2 Projection of points onto cross-section

There exists no closed form solution to the euclidean distance of a given point to a general superellipse. To obtain a simple and computationally inexpensive approximation of the orthogonal projection $\Pi_\theta^{\mathrm{shape}}([p]_{xy})$ of point $p \in \mathbb{R}^3$ onto cross-sectional shape $\varphi(u; \theta)$, defined in Eq (23), we approximated $\varphi(u; \theta)$ by a polygon consisting of n_poly distinct points q_j with linearly spaced parameter values u_j:

$$q_j = \varphi\left(u_j; \theta\right); \quad j = 1, 2, \ldots, n_\mathrm{poly} + 1, \tag{27}$$

$$u_j = \frac{j - 1}{n_\mathrm{poly}} \cdot 2\pi; \quad j = 1, 2, \ldots, n_\mathrm{poly} + 1, \tag{28}$$

where $q_{n_\mathrm{poly}+1} = q_1$.

Using the polygon's edges allowed us to approximate the orthogonal distance of the xy-coordinates of p to the cross-sectional shape:

$$\left|\left|[p]_{xy} - \Pi_\theta^{\text{shape}}([p]_{xy})\right|\right|_2 \approx \min_{j=1,2,\ldots,n_{\text{poly}}} \left|\left|[p]_{xy} - \Pi_{q_j,q_{j+1}}^{\text{edge}}([p]_{xy})\right|\right|_2 \tag{29}$$

Here, $\Pi_{v,w}^{\text{edge}}(s)$ is the orthogonal projection of point s onto the edge between v and w:

$$\Pi_{v,w}^{\text{edge}}(s) = v + l_{v,w}(s) \cdot \frac{(w-v)}{||w-v||_2} \tag{30}$$

with scalar factor

$$l_{v,w}(s) = \max\left\{\min\left\{\frac{w-v}{||w-v||_2} \cdot (s-v)\,,\ ||w-v||_2\right\},\ 0\right\}. \tag{31}$$

Note that Eq (30) and Eq (31) hold for $s, v, w \in \mathbb{R}^2$ and for $s, v, w \in \mathbb{R}^3$ (used in Eq (34) within main text).

4 Smoothing of vessel surface

To obtain a smooth transition of the cross-sectional shapes $\varphi_k(u) := \varphi(u; \theta_k)$ with $k = 1, 2, \ldots, M$, located at positions $z_1 < z_2 < \ldots < z_M$, along the anterior-posterior axis, we defined an interpolating function $\tilde{\theta}$ over the cross-sectional parameters θ_k:

$$\tilde{\theta}\colon [z_1, z_M] \to \mathbb{R}^7, \tag{32}$$

$$\tilde{\theta}(z) := \sum_{k=1}^{M} \theta_k \cdot \hat{\phi}_z(z_k)\,, \tag{33}$$

where $\hat{\phi}_z$ are normalized Gaussian kernel functions. Here, we defined the unnormalized Gaussian kernel functions ϕ_z decaying with distance along the anterior-posterior axis as

$$\phi_z(z_k) := \begin{cases} \exp\left(-\frac{(z_k - z)^2}{2\sigma^2}\right); & z_k \in [\, z - Z_\sigma \cdot \sigma,\ z + Z_\sigma \cdot \sigma\,] \\ 0 \end{cases}, \tag{34}$$

where $\sigma > 0$ and $Z_\sigma > 0$ are tuning parameters controlling the standard deviation of the underlying Gaussian function and the truncation point of the kernel, respectively. We then computed the normalized kernel functions $\hat{\phi}_z$ as

$$\hat{\phi}_z(z_k) = \frac{\phi_z(z_k)}{\sum\limits_{k=1}^{M} \phi_z(z_k)}. \tag{35}$$

Using Eq (33), we obtained cross-sectional shapes $\tilde{\varphi}_k$ with smoothed parameters by

$$\tilde{\varphi}_k(u) := \varphi(u, \tilde{\theta}(z_k)); \quad k = 1, 2, \ldots, M. \tag{36}$$

5 Equidistant points

5.1 Numerical computation of equidistant points

We numerically computed equidistant points on cell contour splines and on superelliptic vessel cross-sections. For a parameterized periodic curve $g\colon [a, b] \to \cdot$ with $g(a) = g(b)$, we

computed n equidistant points $p_1 = g(u_1), p_2 = g(u_2), \ldots, p_n = g(u_n)$ with parameters $u_1, u_2, \ldots, u_n \in [a, b]$ where $u_1 = a$ by numerically solving these minimization problems:

$$u_j = \underset{u \in [a,b]}{\operatorname{argmin}} \left| \Lambda_g(u) - \frac{(j-1)}{n} \Lambda_g(b) \right|; \quad j = 1, 2, \ldots, n, \tag{37}$$

where $\Lambda_g(u)$ is the arc length of $g(u)$ between $t = a$ and $t = u$:

$$\Lambda_g(u) = \int_a^u ||g'(t)||_2 \, \mathrm{d}t. \tag{38}$$

We approximated the arc length integrals $\Lambda_g(u)$ numerically using a fixed-tolerance Gaussian quadrature method.

5.2 Approximation of arc length of superellipse

During the numerical evaluation of the arc length integral of a general superellipse, a singularity is encountered at parameter values $u \in \{0, \pi/2, \pi, 3\pi/2, 2\pi\}$ because the tangent vector there has infinite length. We illustrate this for the (squared) tangent length of the first half of the dorsal superellipse ($0 \leq u \leq \pi/2$):

$$\lim_{u \to 0} ||\varphi'(u)||_2^2 \tag{39}$$

$$= \lim_{u \to 0} \left|\left| \begin{pmatrix} -c\alpha \sin(u) \cos^{\alpha-1}(u) \\ a\alpha \cos(u) \sin^{\alpha-1}(u) \end{pmatrix} \right|\right|_2^2 \tag{40}$$

$$= \underbrace{\left(-c\alpha \frac{\overbrace{\lim_{u \to 0} \sin(u) \cos^{\alpha}(u)}^{\to 0}}{\lim_{u \to 0} \cos(u)} \right)^2}_{\to 0} + \underbrace{\left(a\alpha \frac{\lim_{u \to 0} \cos(u) \sin^{\alpha}(u)}{\underbrace{\lim_{u \to 0} \sin(u)}_{\to 0}} \right)^2}_{\to \infty} \tag{41}$$

$$= \infty \tag{42}$$

and

$$\lim_{u \to \pi/2} ||\varphi'(u)||_2^2 \tag{43}$$

$$= \lim_{u \to \pi/2} \left|\left| \begin{pmatrix} -c\alpha \sin(u) \cos^{\alpha-1}(u) \\ a\alpha \cos(u) \sin^{\alpha-1}(u) \end{pmatrix} \right|\right|_2^2 \tag{44}$$

$$= \underbrace{\left(-c\alpha \frac{\lim_{u \to \pi/2} \sin(u) \cos^{\alpha}(u)}{\underbrace{\lim_{u \to \pi/2} \cos(u)}_{\to 0}} \right)^2}_{\to \infty} + \underbrace{\left(a\alpha \frac{\overbrace{\lim_{u \to \pi/2} \cos(u) \sin^{\alpha}(u)}^{\to 0}}{\lim_{u \to \pi/2} \sin(u)} \right)^2}_{\to 0} \tag{45}$$

$$= \infty. \tag{46}$$

However, the partial arc length of a superellipse in proximity to its singularities can be well approximated by a straight line. For the first half of the dorsal superellipse, we approximated the partial arc lengths near the singularities at $u = 0$ and $u = \pi/2$ by

straight line segments for parameter values $u \in [0, \Delta u]$ and $u \in [\,\pi/2 - \Delta u,\ \pi/2\,]$:

$$\int_0^{\pi/2} ||\varphi(u)||_2 \,\mathrm{d}u \tag{47}$$

$$\approx \left(\int_{\Delta u}^{\pi/2-\Delta u} ||\varphi(u)||_2 \,\mathrm{d}u \right) + ||\varphi(\Delta u) - \varphi(0)||_2 + ||\varphi(\pi/2) - \varphi(\pi/2 - \Delta u)||_2 \,. \tag{48}$$

For each cross-sectional shape, we used the largest value $\Delta u > 0$ such that the angle between the tangent vector *at* any singularity and the corresponding tangent vector *near* that singularity with parameter value shifted by Δu was below 1°. Further ideas for approximations of the superelliptic arc length integral and equidistant sampling of superellipses can be found in [1].

6 Projection of endothelial cell contours onto vessel surface

We projected each endothelial cell contour spline $s_{\mathrm{transf},i}$ separately onto the estimated vessel surface. For each cell i, we split the cell's spline into segments between subsequent (equidistant) points $p, p^+ \in \mathrm{EC}_{\mathrm{transf},i}$. To simplify notation, we subsequently only consider the case where the spline parameter value $[p]_u$ of point p on the spline is lower than the parameter value $[p^+]_u$ of its successor p^+. Note however that cases where $[p]_u > [p^+]_u$ also have to be taken into account due to the splines' periodicity. We then defined the spline segment between points p and p^+ as the restriction of $s_{\mathrm{transf},i}$ to the parameter domain $\left[\,[p]_u\,, [p^+]_u\,\right]$. Next, each of these spline segments was projected onto the grid approximation of the vessel surface (see S4 Fig).

For the spline segment defined by the points p and p^+, we first identified the set of cross-sectional planes intersecting this spline segment, i.e., the set of z_k such that either $[p]_z \leq z_k \leq [p^+]_z$ or $[p]_z \geq z_k \geq [p^+]_z$ (see S4A Fig). We then projected the spline segment onto each of the cross-sectional shapes on the intersected planes. To project the spline segment onto the cross-sectional shape at z_k, we first applied a bisection method to identify the parameter $u^* \in \left[\,[p]_u\,, [p^+]_u\,\right]$ such that $s_{\mathrm{transf},i}(u^*)$ was located at the intersection of the spline segment and the cross-sectional plane, i.e., $[s_{\mathrm{transf},i}(u^*)]_z \approx z_k$. We then approximated the projection of $s_{\mathrm{transf},i}(u^*)$ onto the cross-sectional shape at z_k by the nearest neighbor of $s_{\mathrm{transf},i}(u^*)$ among the points $q \in \mathrm{CS}_k$ on the local cross-sectional shape (see S4B Fig). If two consecutive points of the cell contour were projected onto the same cross-sectional shape, all points on that shape lying on the shortest path between the two projections were also added to the projected contour.

As we defined spline segments via a dense sequence of equidistant points, we assumed in the projection method that each parameter interval $\left[\,[p]_u\,, [p^+]_u\,\right]$ was narrow enough that the z-coordinates of points on the spline were monotonically increasing or decreasing within this interval. Independently of this assumption, we found during an intermediate analysis that the projection method could result in self-intersections of the projected cell contours. However, in case of the data sets analyzed in this article, all self-intersections disappeared when we either corrected cell contour annotations or increased the number of points used to approximate cross-sectional shapes.

7 Triangulation within projected endothelial cell contours

To obtain a triangulated surface of a single cell we proceeded as follows: First, we triangulated the entire vessel surface between the minimal and maximal z-coordinate of the cell (see S5 Fig). This triangulation was performed between each pair of neighboring vessel cross-sections. For two given neighboring vessel cross-sections, we collected all edges of the projected cell contour connecting these two cross-sections. We then triangulated the entire vessel surface between these two cross-sections ensuring that each of the collected cell contour edges was contained in a triangle. Secondly, we extracted the subset of triangles lying within the projected cell contour (see S6 Fig). We now explain the steps of this triangulation algorithm in detail.

7.1 Collection of edges between neighboring cross-sections

All edges E_{ik} between points on cross-sections k and $(k+1)$ are collected that are part of the projected cell contour of cell i:

$$E_{ik} = \Big\{ \ (p,q) \in \mathrm{CS}_k \times \mathrm{CS}_{k+1} : \ p,q \in \mathrm{EC}_{\mathrm{proj},i} \ \wedge \ \big(q = p^+ \vee p = q^+\big) \ \Big\}. \tag{49}$$

Note that here the successor relationship $(+)$ refers to the (projected) cell contour, i.e., for each $e = (p,q) \in E_{ik}$ there exists an edge (p,q) or (q,p) in $\mathrm{EC}_{\mathrm{proj},i}$.

The edges $e_j = (p_j, q_j) \in E_{ik}$ with $j = 1, 2, \ldots, n_{ik}$ are then sorted as follows: Starting at the points p_1, q_1 on cross-sectional shape k and $(k+1)$, respectively, each cross-sectional shape is traversed exactly once in direction of increasing cross-section parameter value. During this traversal, on each cross-sectional shape the points p_j, q_j are encountered exactly once in their sorted order. First p_2, q_2 are encountered, then p_3, q_3 etc. until $p_{n_{ik}}$, $q_{n_{ik}}$ are reached. We denote this sorted sequence of edges of the projected cell contour of cell i between cross-sections k and $(k+1)$ as

$$\tilde{E}_{ik} = \Big((p_1, q_1)\,, (p_2, q_2)\,, \ldots, (p_{n_{ik}}, q_{n_{ik}})\,, (p_{n_{ik}+1}, q_{n_{ik}+1}) \Big), \tag{50}$$

where $(p_{n_{ik}+1}, q_{n_{ik}+1}) = (p_1, q_1)$ is added to account for cross-sectional periodicity. Mathematically, the above sorting fulfills these two conditions:

$$[p_1]_u \le [p_2]_u \le \cdots \le [p_{n_{ik}}]_u, \tag{51}$$
$$[q_j]_u - [q_{j-1}]_u \le [q_{j+1}]_u - [q_{j-1}]_u \pmod{2\pi}; \quad j = 2, 3, \ldots, n_{ik}. \tag{52}$$

Note that the parameter value u refers to the cross-sectional shape $\tilde{\varphi}_k(u)$ and not to a cell contour spline.

7.2 Paths on cross-sectional shape between two edges

For each pair of subsequent edges $e_j = (p_j, q_j) \in \tilde{E}_{ik}$ and $e_{j+1} = (p_{j+1}, q_{j+1}) \in \tilde{E}_{ik}$ with $j = 1, 2, \ldots n_{ik}$, paths are constructed on the corresponding cross-sectional shapes k and $(k+1)$ between points p_j and p_{j+1} and between points q_j and q_{j+1}, respectively (see also S5A Fig). These paths define the order of points used to triangulate the vessel surface between edges e_j and e_{j+1}. Path $P(v, v')$ between $v, v' \in \mathrm{CS}_k$ going in direction of increasing parameter value is computed as

$$P(v,v') = \begin{cases} (v, v^+, \ldots, (v')^-, v')\,; & [v']_u > [v]_u \\ (v', (v')^+, \ldots, v^-, v)\,; & [v']_u < [v]_u \end{cases} . \tag{53}$$

In the case of $[v']_u = [v]_u$ with $v, v' \in \mathrm{CS}_k$, whenever the corresponding path on the neighboring cross-section CS_{k+1} is shorter or longer than its complementary path on CS_{k+1}, path (v) or path $(v, v^+, \dots, v^-, v)$ has to be used, respectively.

7.3 Triangulation between two paths on neighboring cross-sections

Consider the two cross-sectional paths $P = (v_1, v_2, \dots, v_n)$ and $P' = (w_1, w_2, \dots, w_m)$ with $m \geq n$ on neighboring cross-sectional shapes. In a first step, each point from P is connected to two neighboring points from P' with a triangle. In a second step, the surface between the two paths is closed by connecting each point from P' to two neighboring points from P with a triangle. S5B Fig shows an example how the triangulated cell surface area between two paths on neighboring cross-sections is constructed.

In the first step, $(m - 1)$ triangles are added where each triangle consists of exactly one point $v_j \in P$ and two neighboring points $w_{j'}, w_{j'+1} \in P'$. This ensures that each edge $(w_{j'}, w_{j'+1})$ of P' is contained in exactly one triangle. Within this set of triangles, at least $N_{\mathrm{tri}} \in \mathbb{N}_0$ triangles contain $v_j \in P$ with $j = 1, 2, \dots, n$, where

$$N_{\mathrm{tri}} = \left\lfloor \frac{m-1}{n} \right\rfloor . \tag{54}$$

As a result,

$$\bar{N}_{\mathrm{tri}} = (m-1) - (N_{\mathrm{tri}} \cdot n) \ \in \mathbb{N}_0 \tag{55}$$

triangles remain to be distributed.

To facilitate expressing the set of triangles containing $v_j \in P$, we introduce an auxiliary vector ζ, where ζ_j counts the number of triangles containing $v_j \in P$ and two neighboring points $w_{j'}, w_{j'+1} \in P'$:

$$\zeta = \Big(\underbrace{(N_{\mathrm{tri}}+1), \dots, (N_{\mathrm{tri}}+1)}_{\lceil \bar{N}_{\mathrm{tri}}/2 \rceil}, \underbrace{N_{\mathrm{tri}}, \dots, N_{\mathrm{tri}}}_{(n-\bar{N}_{\mathrm{tri}})}, \underbrace{(N_{\mathrm{tri}}+1), \dots, (N_{\mathrm{tri}}+1)}_{\lfloor \bar{N}_{\mathrm{tri}}/2 \rfloor} \Big)^{\top} \in \mathbb{N}_0^n. \tag{56}$$

We denote the cumulative sums of ζ as Z_j:

$$Z_j \coloneqq \sum_{j'=1}^{j} \zeta_{j'}; \quad j = 0, 1, \dots, n. \tag{57}$$

For each point $v_j \in P$ with $j = 1, 2, \dots, n$, we are now able to define the triangles $\Delta_{jj'}$ containing v_j and two points $w_{j'}, w_{j'+1} \in P'$:

$$\Delta_{jj'} \coloneqq (v_j, w_{j'}, w_{j'+1}); \quad j' = Z_{j-1}+1,\ Z_{j-1}+2,\ \dots,\ Z_j. \tag{58}$$

In a second step, the surface between the two paths is closed via construction of $(n-1)$ triangles Δ_j consisting of two points $v_j, v_{j+1} \in P$ and a point $w_{Z_j+1} \in P'$:

$$\Delta_j \coloneqq \big(v_j, v_{j+1}, w_{Z_j+1}\big); \quad j = 1, 2, \dots, n-1. \tag{59}$$

This second step ensures that each edge (v_j, v_{j+1}) of P is contained in exactly one triangle.

7.4 Refinement of triangulation

To refine the triangulation between two subsequent edges $e_j = (p_j, q_j) \in \tilde{E}_{ik}$ and $e_{j+1} = (p_{j+1}, q_{j+1}) \in \tilde{E}_{ik}$ with $j = 1, 2, \dots n_{ik}$, additional edges between points on

$P(p_j, p_{j+1})$ and $P(q_j, q_{j+1})$ that connect points with their nearest neighbor on the other cross-section are included in the triangulation (see also S5C Fig). For $v \in \{p_j, p_{j+1}\}$ it is checked whether the cross-sectional path $P(q_j, q_{j+1})$ includes the nearest neighbor $\Pi_{k+1}(v)$ of v on the cross-sectional shape $(k+1)$. Here, the nearest neighbor $\Pi_{k+1}(v)$ on cross-section $(k+1)$ of point $v \in \mathrm{CS}_k$ is

$$\Pi_{k+1}(v) = \underset{w \in \mathrm{CS}_{k+1}}{\operatorname{argmin}} \, ||v - w||_2 \,. \tag{60}$$

If $\Pi_{k+1}(v) \in P(q_j, q_{j+1})$, the edge $(v, \Pi_{k+1}(v))$ is included in the triangulation, i.e., the triangulation between edges e_j, e_{j+1} is split into two smaller triangulations. The triangulation refinement for $w \in \{q_j, q_{j+1}\}$ is performed in an analogous manner.

7.5 Extraction of cell mesh

Starting from the triangulation $\mathbf{T}$ of the complete vessel surface within the cell's minimal and maximal z-coordinates, a mesh $T_i \subset \mathbf{T}$, which only contains the cell's surface, is extracted in a semi-automatic manner (see also S6 Fig): Starting with an initial triangle $\Delta \in \mathbf{T}$ within the cell's surface, neighboring triangles are iteratively added to the mesh T_i, provided they are located within the (projected) cell contour. In most cases a suitable initial triangle is found by projecting the (component-wise) arithmetic mean $\bar{p}_i$ of the projected cell contour $\mathrm{EC}_{\mathrm{proj},i}$ onto the nearest neighbor $q^* \in \mathrm{CS}$ on the grid approximation of the vessel surface and then choosing any triangle $\Delta \in \mathbf{T}$ with $q^* \in \Delta$. Here,

$$\bar{p}_i = \frac{1}{n_{\mathrm{proj},i}} \sum_{p \in \mathrm{EC}_{\mathrm{proj},i}} p, \tag{61}$$

$$q^* = \underset{q \in \mathrm{CS}}{\operatorname{argmin}} \, ||\bar{p}_i - q||_2 \,. \tag{62}$$

However, q^* can be located outside the cell surface. In this case, an initial triangle has to be provided manually.

Further details on the cell's mesh extraction can be found in S2 Algorithm. The input to the algorithm is the set $E_{\mathrm{contour},i}$ of (undirected) edges of the projected cell contour of cell i:

$$E_{\mathrm{contour},i} = \Big\{ \{v, w\} : \; v, w \in \mathrm{EC}_{\mathrm{proj},i} \; \wedge \; \big(v^+ = w \vee w^+ = v\big) \Big\}. \tag{63}$$

Here, the successor relationship refers to the projected cell contour.

8 Elongation of endothelial cells

8.1 Cell surface bounding box

To quantify elongation of a cell surface in the direction of flow, we first computed a cell surface bounding box covering the cell's extension within the cross-sectional plane and the cell's length in the direction of flow (see also Fig 6A within main text). To compute this box, we projected all points of the cell's mesh onto the cross-sectional (xy-)plane and then determined the smallest angular range within the cross-sectional plane that contains all these points. For each point on the grid approximation of the vessel surface, we checked whether it lies inside this angular range when it is projected onto the xy-plane. We included all vessel surface points in the bounding box that lie within this angular range and also have a z-coordinate within the cell mesh's minimal and maximal z-coordinates. Finally, we connected the bounding box's points by triangulation.

S2 Algorithm: Cell mesh extraction

input : cell contour edges E_{contour}, starting triangle Δ, vessel surface triangulation $\mathbf{T}$

output : triangulation subset within cell contour $T \subset \mathbf{T}$

$T = \{\Delta\}$

$T_{\text{reject}} = \varnothing$

`// Contour edges contained in cell surface mesh`

$E_{\text{seen}} = \{\, e \in E_{\text{contour}} : |\Delta \cap e| = 2 \,\}$

put Δ into Q

while $Q \neq \varnothing$ **do**

  $\Delta \leftarrow$ get triangle from Q

  `// Neighboring triangles of Δ share an edge`

  $N_\Delta = \{\, \Delta' \in \mathbf{T} : |\Delta' \cap \Delta| = 2 \,\}$

  for $\Delta' \in N_\Delta$ **do**

    if $\Delta' \notin T \wedge \Delta' \notin T_{reject}$ **then**

      `// Check if the triangle lies outside the cell contour`

      for $e \in E_{seen}$ **do**

        if $|e \cap \Delta'| = 2$ **then**

          `// Reject triangle`

          $T_{\text{reject}} \leftarrow T_{\text{reject}} \cup \Delta'$

      `// Triangles inside the cell contour are added to the cell surface mesh`

      if Δ' *not rejected* **then**

        `// Document all contour edges contained in the triangle`

        $E_{\text{seen}} \leftarrow E_{\text{seen}} \cup \{\, e \in E_{\text{contour}} : |\Delta' \cap e| = 2 \,\}$

        `// Add triangle to cell surface mesh`

        $T \leftarrow T \cup \Delta'$

        put Δ' into Q

We now explain in detail how this bounding box can be constructed from a cell surface mesh.

For each cell i, its surface mesh contains a set V_i of points:

$$V_i = \{\, p \in \mathrm{CS} : \exists \Delta \in T_i \colon p \in \Delta \,\} \subset \mathbb{R}^3. \tag{64}$$

To define the cell surface bounding box, we introduce the function ψ that measures the angle between the xy-coordinates of $p \in \mathbb{R}^3$ and the x-axis:

$$\psi\colon \mathbb{R}^3 \to [0, 2\pi), \tag{65}$$

$$\psi(p) = \mathrm{atan2}([p]_{xy}), \tag{66}$$

where $\mathrm{atan2}(q)$ returns the angle between $q \in \mathbb{R}^2$ and the x-axis in the range $[0, 2\pi)$. Thus, the set Ψ_i consisting of the angles of all points that are contained in the mesh of cell i is:

$$\Psi_i = \{\, \psi(p) : p \in V_i \,\}. \tag{67}$$

We denote the sorted angles as

$$\Psi_{\mathrm{sort},i} = (\psi_1, \psi_2, \ldots, \psi_{n+1}), \tag{68}$$

where $\psi_j \in \Psi_i$ for all $j = 1, 2, \ldots, n+1$ and $\psi_1 \leq \psi_2 \leq \ldots \leq \psi_n$. Here, $\psi_{n+1} = \psi_1$ is appended to account for periodicity within the vessel cross-section.

The largest distance between two neighboring angles of the sorted sequence $\Psi_{\mathrm{sort},i}$ defines an angular gap $(\psi_{j^*}, \psi_{j^*+1})$ that excludes all angles of points of the cell surface mesh. Mathematically, the index j^* is computed as

$$j^* = \underset{j=1,2,\ldots,n}{\mathrm{argmax}} \Big((\psi_{j+1} - \psi_j) \pmod{2\pi} \Big). \tag{69}$$

The set $V_{\mathrm{angle},i}$ of all points on vessel cross-sections that fall outside this angular gap is

$$V_{\mathrm{angle},i} = \begin{cases} \{\, p \in \mathrm{CS} : \psi(p) \leq \psi_{j^*} \,\vee\, \psi(p) \geq \psi_{j^*+1} \,\}; & j^* = 1, 2, \ldots, n-1 \\ \{\, p \in \mathrm{CS} : \psi_1 \leq \psi(p) \leq \psi_n \,\}; & j^* = n \end{cases}. \tag{70}$$

Additionally, the set $V_{\mathrm{z},i}$ of all points on vessel cross-sections located within the cell mesh's range of z-coordinates is:

$$V_{\mathrm{z},i} = \{\, p \in \mathrm{CS} : z_{\mathrm{min},i} \leq [p]_z \leq z_{\mathrm{max},i} \,\}, \tag{71}$$

where $z_{\mathrm{min},i}$ and $z_{\mathrm{max},i}$ are the cell mesh's minimal and maximal z-coordinate, respectively:

$$z_{\mathrm{min},i} = \min_{p \in V_i} [p]_z, \tag{72}$$

$$z_{\mathrm{max},i} = \max_{p \in V_i} [p]_z. \tag{73}$$

The points $V_{\mathrm{bb},i}$ of the bounding box can then be defined as the intersection of these two sets:

$$V_{\mathrm{bb},i} = V_{\mathrm{angle},i} \cap V_{\mathrm{z},i}. \tag{74}$$

Finally, a triangulation $T_{\mathrm{bb},i}$ of the bounding box is constructed. This triangulation contains all the projected cell contour edges and also all edges between neighboring points of the box's boundary.

8.2 Computation of elongation

Using the triangulated cell surface bounding box, we quantified cell elongation as the ratio of the box's extension in flow (z-)direction to its extension within the cross-sectional plane (see Eq (75)). As the extension within the cross-sectional plane varied along the vessel's anterior-posterior axis, we used the cell's mean extension within the cross-sectional plane. This mean extension can be derived from the surface area of the cell's bounding box and its extension in the direction of flow (see Eq (76)). Thus, elongation elong_i of cell i is computed as

$$\text{elong}_i := \frac{\text{extension of } T_{\text{bb},i} \text{ in the direction of flow}}{\text{mean extension of } T_{\text{bb},i} \text{ within cross-sectional plane}} \tag{75}$$

$$= \frac{z_{\max,i} - z_{\min,i}}{\frac{\text{area of } T_{\text{bb},i}}{z_{\max,i} - z_{\min,i}}} \tag{76}$$

$$= \frac{\left(z_{\max,i} - z_{\min,i}\right)^2}{\text{area of } T_{\text{bb},i}}. \tag{77}$$

9 Classification of endothelial cells

To study whether the location of ECs within the DA influenced their morphology, we classified cells by their location within the DA's cross-sectional plane. For this, we first split the cross-sectional plane into octants and used them to divide the entire DA surface into dorsal, ventral, left and right sectors. Next, we computed each cell's partial surface area per plane sector. Finally, we classified each cell by the plane sector containing the majority of its surface area.

9.1 Plane octants

To allow classification of ECs, we employed octants oct_m in the cross-sectional (xy-)plane that are constant along the anterior-posterior (z-)axis:

$$\text{oct}_m := \left\{ q \in \mathbb{R}^2 : (m-1)\pi/4 \leq \text{atan2}(q) \leq m\pi/4 \right\}; \quad m = 1, 2, \ldots, 8, \tag{78}$$

where $\text{atan2}(q)$ returns the angle between $q \in \mathbb{R}^2$ and the x-axis in the range $[0, 2\pi)$. Note that these octants were also applied during the estimation of cross-sectional shapes.

9.2 Plane sectors

The plane octants allowed us to define sectors of the vessel surface as sets of points that are located in different angular sectors when projected into the xy-plane (see also Fig 6B within main text):

$$\text{dorsal sector} := \left\{ p \in \mathbb{R}^3 : [p]_{xy} \in \text{oct}_2 \cup \text{oct}_3 \right\}, \tag{79}$$

$$\text{left sector} := \left\{ p \in \mathbb{R}^3 : [p]_{xy} \in \text{oct}_4 \cup \text{oct}_5 \right\}, \tag{80}$$

$$\text{ventral sector} := \left\{ p \in \mathbb{R}^3 : [p]_{xy} \in \text{oct}_6 \cup \text{oct}_7 \right\}, \tag{81}$$

$$\text{right sector} := \left\{ p \in \mathbb{R}^3 : [p]_{xy} \in \text{oct}_1 \cup \text{oct}_8 \right\}, \tag{82}$$

where oct_m with $m = 1, 2, \ldots, 8$ are the octants in the xy-plane (see Eq (78)). Note that these sectors are identical along the anterior-posterior axis.

9.3 Partial cell surfaces

To then classify an EC, we computed its partial surface areas lying in either of the cross-sectional sectors defined in Eq (79) – Eq (82). For this, we first clipped the cell's mesh with a set of planes. Clipping removes triangles of a mesh on one side of the provided plane. Triangles that cross this plane are first split into smaller triangles. To obtain the cell's dorsal surface area, we first clipped the cell mesh by a plane with origin $(0, 0, 0)^\top$ and normal vector $(-1, -1, 0)^\top$. We then clipped the resulting mesh with a plane with origin $(0, 0, 0)^\top$ and normal vector $(1, -1, 0)^\top$. The cell's left, ventral and right surface areas were obtained in an analogous manner. We performed these operations using pyvista [2].

9.4 Simple cell classification

In the main analysis, we classified cells by the plane sectors containing the majority of their cell surface area: If a cell's partial surface area lying in the dorsal sector was greater than 50 % of its total surface area, we classified the cell as a dorsal cell; ventral cells were analogously classified. We did not distinguish between left and right cells. Thus, if a cell's partial surface area lying in *either* the left *or* right sector was greater than 50 % of its total surface area, we classified the cell as a left/right cell.

9.5 Detailed cell classification

In a follow-up analysis, we compared the morphology of subpopulations of dorsal, ventral or left/right cells. For this, we employed a finer classification: We labeled dorsal cells whose partial surface area in the dorsal sector was greater than 75 % of their total area as *exclusively* dorsal cells (Ds). Cells whose partial cell surface area in the dorsal sector was higher than 50 % of their total surface area but *maximally* 75 %, were labeled as dorsal-left/right cells (D-LRs). These cells wrapped around a larger fraction of the DA than Ds. Exclusively ventral cells (Vs) and ventral cells wrapping around the DA's left or right side (V-LRs) were defined in an analogous manner.

Again, we did not distinguish between left and right cells: We labeled cells whose partial surface area in *either* the left *or* right sector was greater than 75 % of their total area as *exclusively* left/right cells (LRs). Cells whose partial surface area in either the left or right sector was higher than 50 % of their total surface area but *maximally* 75 % were considered to be wrapping around the DA. If their partial cell surface area in the dorsal sector was higher than their partial area in the ventral sector, we labeled them as left/right-dorsal cells (LR-Ds). Instead, if their partial area in the vector sector was higher, they were labeled as left/right-ventral cells (LR-Vs).

10 Criteria for the choice of tuning parameter values

Our novel mathematical approach is fine-tuned by 13 parameters (see also S2 Table). These tuning parameters can be broadly categorized as (1) controlling the overall *precision* of cell contour description or vessel surface reconstruction, (2) capturing the *complexity* of the underlying experimental data and (3) directly controlling the *local quality of estimated* vessel cross-sections.

10.1 Preprocessing of fluorescent images

For each of the 5 tuning parameters that control the preprocessing of manually annotated EC contours, we applied the *same* value to all wild-type and Endoglin-deficient embryos at both 48 hpf and 72 hpf. To ensure that these values were suitable for all data sets, i.e.,

embryos at either time point, we visually compared the results of contour preprocessing with different parameter values. In a conservative manner, we chose values for the two *data-complexity* parameters, (1) the radius r and (2) the length extension Δh that together define cylinders around cell contour edges during cell contour enrichment by neighbors: We first identified ranges for both r and Δh containing small enough values of these parameters such that for each cell only those close-by contour segments, which were part of its cell-cell contacts, were integrated into its contour. Within these ranges, we chose large enough values for r and Δh such that the combined information on a cell-cell contact of any two adjacent cells was integrated into both contours. We chose a sufficiently high value for (3) the *precision* parameter $n_{\text{interp,rel}}$ that controls the relative number of points linearly interpolated on edges of enriched cell contours such that long straight contour segments were well-preserved during cell contour approximation by smoothing splines in the subsequent step. For (4) the *precision* parameter ϵ_{spline} controlling the upper bound on the allowed mean squared distance of fitted contour splines to enriched cell contours, our chosen value was small enough that splines closely fitted to the enriched EC contours and large enough to smooth sharp contour bends. Finally, we chose a high value for (5) the *precision* parameter $n_{\text{spline,rel}}$ that controls the relative number of equidistant points computed on cell contour splines such that each cell contour spline was approximated by a dense sequence of points.

10.2 Vessel surface reconstruction

For each of the 8 tuning parameters that control the vessel surface reconstruction, we applied the *same* value to all wild-type and Endoglin-deficient embryos at both 48 hpf and 72 hpf. To accurately reconstruct local vessel geometry, we chose high values for *precision* parameters: (1) the relative number M_{rel} of vessel cross-sections estimated along the anterior-posterior axis, (2) the number n_{poly} of points used to approximate the orthogonal projection of cell contour points onto the locally estimated shape and (3) the relative number $n_{\text{cross,rel}}$ of points computed on each smoothed cross-sectional shape. We fixed (4) *local-estimation quality* parameter $Z_\omega = 4$ that controls the distance of the truncation point in either direction from the mean value of the Gaussian function underlying the weight function employed during the estimation of cross-sectional shapes. Additionally, we fixed (5) *local-estimation quality* parameter $Z_\sigma = 4$ that controls the distance of the truncation point in either direction from the mean value of the underlying Gaussian function employed during smoothing of vessel cross-sections along the axis.

The presented vessel surface reconstruction method balances the locality of estimated cross-sectional shapes, the physiological plausibility of these shapes and the overall smoothness of the vessel surface. These trade-offs are controlled by three *local-estimation quality* parameters: (6) the minimal number n_{oct} of points with non-zero weight per plane octant during estimation of local cross-sectional shapes, (7) the upper bound λ on the allowed relative deviation of the locally estimated cross-sectional shape from the mean shape and (8) the standard deviation σ of the underlying Gaussian function employed during smoothing of cross-sectional shapes. Hence, we performed a systematic parameter search: We first chose a value for n_{oct} while not constraining the deviation of the local cross-sectional shape from the mean shape (Eq (18) within main text) and not smoothing the estimated cross-sectional shapes along the vessel axis. After fixing n_{oct}, we chose a value for λ, again without smoothing the estimated cross-sectional shapes along the vessel axis. Once λ was also identified, we finally chose σ. For each tested combination of values of n_{oct}, λ and σ, we measured goodness of fit by computing the distances between the manually annotated EC contours and their projections onto the vessel surface using Eq (36) (main text). We computed a lower bound of these contour distances by using $n_{\text{oct}} = 1$ during unconstrained estimation without any subsequent smoothing. An upper bound of these contour distances was computed by using the mean

shape along the entire vessel axis. Additionally, we visually inspected the physiological plausibility of the estimated shapes and the smoothness of the resulting vessel surface for each tested combination of values of n_{oct}, λ and σ. In large sectors of the cross-sectional plane that only contained points with low weights during the shape's estimation, a physiologically plausible cross-sectional shape would either locally resemble the mean shape or closely match those points with low weights.

11 Unrolling of endothelial cell surfaces

We unrolled EC surfaces in a two-step procedure: First, we cut the vessel surface along the anterior-posterior axis and hereby obtained a two-dimensional representation of the entire vessel surface. Next, we reconnected all EC surfaces that were cut during unrolling. Note that unrolling of a vessel surface with variable cross-sectional shapes along the anterior-posterior axis would result in cartographic distortions. Hence, before unrolling we re-estimated each vessel surface using a constant cross-sectional shape, namely the mean shape $\bar{\varphi}$, by setting $\lambda = 0\,\%$ during cross-section estimation.

When estimating vessel cross-sections using the mean shape only, the grid approximation of the vessel surface is simplified: the coordinates of (equidistant) points $p_j \in \text{CS}_k$ on the cross-sectional shape $\tilde{\varphi}_k = \bar{\varphi}$ at z_k are given as

$$p_j = \Big([\bar{\varphi}(u_j)]_x,\ [\bar{\varphi}(u_j)]_y,\ z_k \Big); \quad j = 1, 2, \ldots, n_k \tag{83}$$

(compare with Eq (20) within main text). To unroll point p_j, we define an unrolling function $\bar{\varphi}_{\text{unroll}}$ that maps the point's xy-coordinates to the point's associated partial arc length $\Lambda_{\bar{\varphi}}\left([p_j]_u\right)$ between $u = 0$ and $u = [p_j]_u$ (see Eq (38)) while preserving the point's z-coordinate:

$$\bar{\varphi}_{\text{unroll}} \colon \mathbb{R}^3 \to \mathbb{R}^2 \tag{84}$$

$$\bar{\varphi}_{\text{unroll}}(p_j) = \Big(\Lambda_{\bar{\varphi}}([p_j]_u),\ [p_j]_z \Big)^\top . \tag{85}$$

We denote the coordinates of unrolled points $q_j = \bar{\varphi}_{\text{unroll}}(p_j)$ as

$$q_j = \left([q_j]_l\,, [q_j]_z \right)^\top . \tag{86}$$

Note that unrolling cuts the vessel surface along the anterior-posterior axis. This disconnects contour segments in a subset of cells. We reconnected these contours by moving a subset of their points q_j across the periodicity boundary of the unrolled vessel surface, i.e., by altering the unrolled points' l-coordinates:

$$[q_j]_l \leftarrow [q_j]_l \pm \Lambda_{\bar{\varphi}}(2\pi). \tag{87}$$

12 Weighted statistics

To allow comparison of our computed morphometric measurements in ECs with literature-reported mean values, we computed weighted means μ_{w} over subsets J of all analyzed data sets, e.g., wild-type embryos at 48 hpf. Each data set $m \in J$ contains a set I_m of ECs with projection distances that are not higher than the maximal annotation uncertainty. We weighted the value $x_{m,i}$ of a morphometric measurement in cell $i \in I_m$ of data set $m \in J$ by the number $|I_m|$ of ECs in the same data set:

$$\mu_{\text{w}} = \frac{\sum\limits_{m \in J} \sum\limits_{i \in I_m} |I_m| \cdot x_{m,i}}{\sum\limits_{m \in J} \sum\limits_{i \in I_m} |I_m|} = \frac{\sum\limits_{m \in J} |I_m| \sum\limits_{i \in I_m} x_{m,i}}{\sum\limits_{m \in J} |I_m|^2} \tag{88}$$

To quantify overall variability in the subsets J, we additionally computed a weighted sample variance σ_{w}^2 using the same weights as above:

$$\sigma_{\mathrm{w}}^2 = \frac{\sum\limits_{m\in J}\sum\limits_{i\in I_m} |I_m| \cdot (x_{m,i} - \mu_{\mathrm{w}})^2}{\left(\sum\limits_{m\in J}\sum\limits_{i\in I_m} |I_m|\right) - 1} = \frac{\sum\limits_{m\in J} |I_m| \sum\limits_{i\in I_m} (x_{m,i} - \mu_{\mathrm{w}})^2}{\left(\sum\limits_{m\in J} |I_m|^2\right) - 1} \tag{89}$$

Using Eq (89) allowed us to compute a weighted standard deviation as

$$\sigma_{\mathrm{w}} = \sqrt{\sigma_{\mathrm{w}}^2}. \tag{90}$$

We defined weighted statistics over geometric measurements in an analogous manner, i.e., values were weighted by the number of vessel cross-sections in the same data set. Here, vessel cross-sections where only one cell was located within a distance of 0.5 µm to the cross-sectional plane were excluded.

References

1. Pilu M, Fisher R. Equal-Distance Sampling of Superellipse Models. In: Proceedings of the British Machine Vision Conference. BMVA Press; 1995. p. 26.1–26.10.

2. Sullivan CB, Kaszynski A. PyVista: 3D plotting and mesh analysis through a streamlined interface for the Visualization Toolkit (VTK). Journal of Open Source Software. 2019;4(37):1450. doi:10.21105/joss.01450.
