# Supplementary material for: Novel mathematical approach to accurately quantify 3D endothelial cell morphology and vessel geometry based on fluorescently marked endothelial cell contours: Application to the dorsal aorta of wild-type and Endoglin-deficient zebrafish embryos
Source: PLoS Comput Biol. 2024 Aug 30;20(8):e1011924. doi: 10.1371/journal.pcbi.1011924 (PMC11392406; doi:10.1371/journal.pcbi.1011924)

S1 Tutorial: Inspecting endothelial cell surface meshes using Paraview[1]

Novel mathematical approach to accurately quantify 3D endothelial cell morphology and vessel geometry based on fluorescently marked endothelial cell contours: Application to the dorsal aorta of wild-type and Endoglin-deficient zebrafish embryos

Daniel Seeler, Nastasja Grdseloff, Claudia Jasmin Rödel, Charlotte Kloft, Salim Abdelilah-Seyfried, Wilhelm Huisinga

[1] Ayachit, Utkarsh, The ParaView Guide: A Parallel Visualization Application, Kitware, 2015, ISBN 9781930934306 (www.paraview.org)

Installing Paraview

- Download and install a suitable version of Paraview for your operating system from

https://www.paraview.org/download/

[This tutorial is based on Paraview 5.11.2 on an Intel-based MacBook with macOS 12.7]

Endothelial cell surface (mesh) files

Files:

- There is one .vtmb file for each data set (embryo at a fixed time point)
- Each .vtmb file uses the .vtp files found in the folder with the same name

Each data set contains:

1) the manually annotated endothelial cell contours; their coordinates have been transformed into the estimated coordinate system
2) the endothelial cell surface meshes estimated from these contours

Setting up visualization

Opening the first file

1) Open .vtmb file.

2) Click on closed eye.

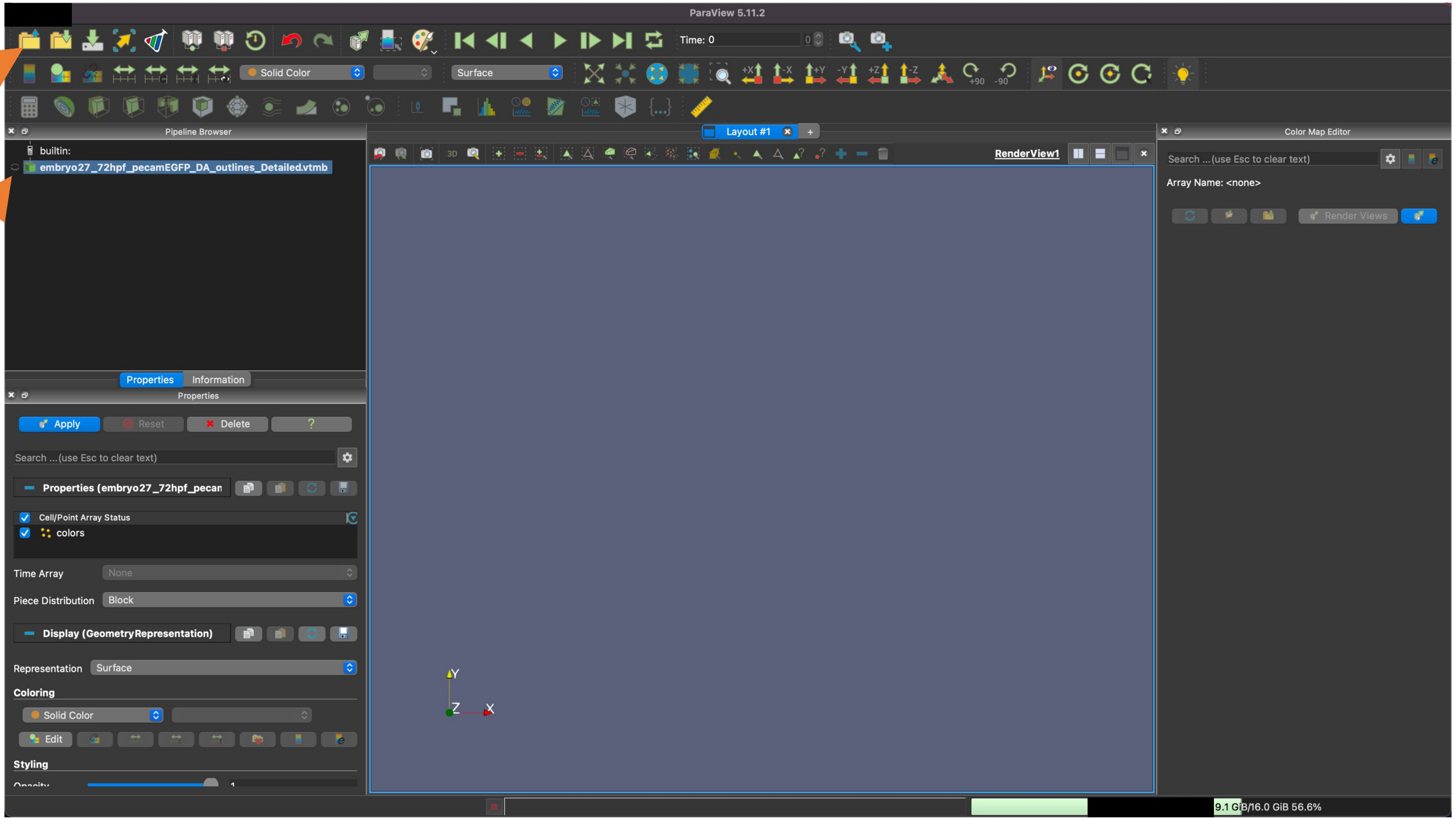

Coloring cells I

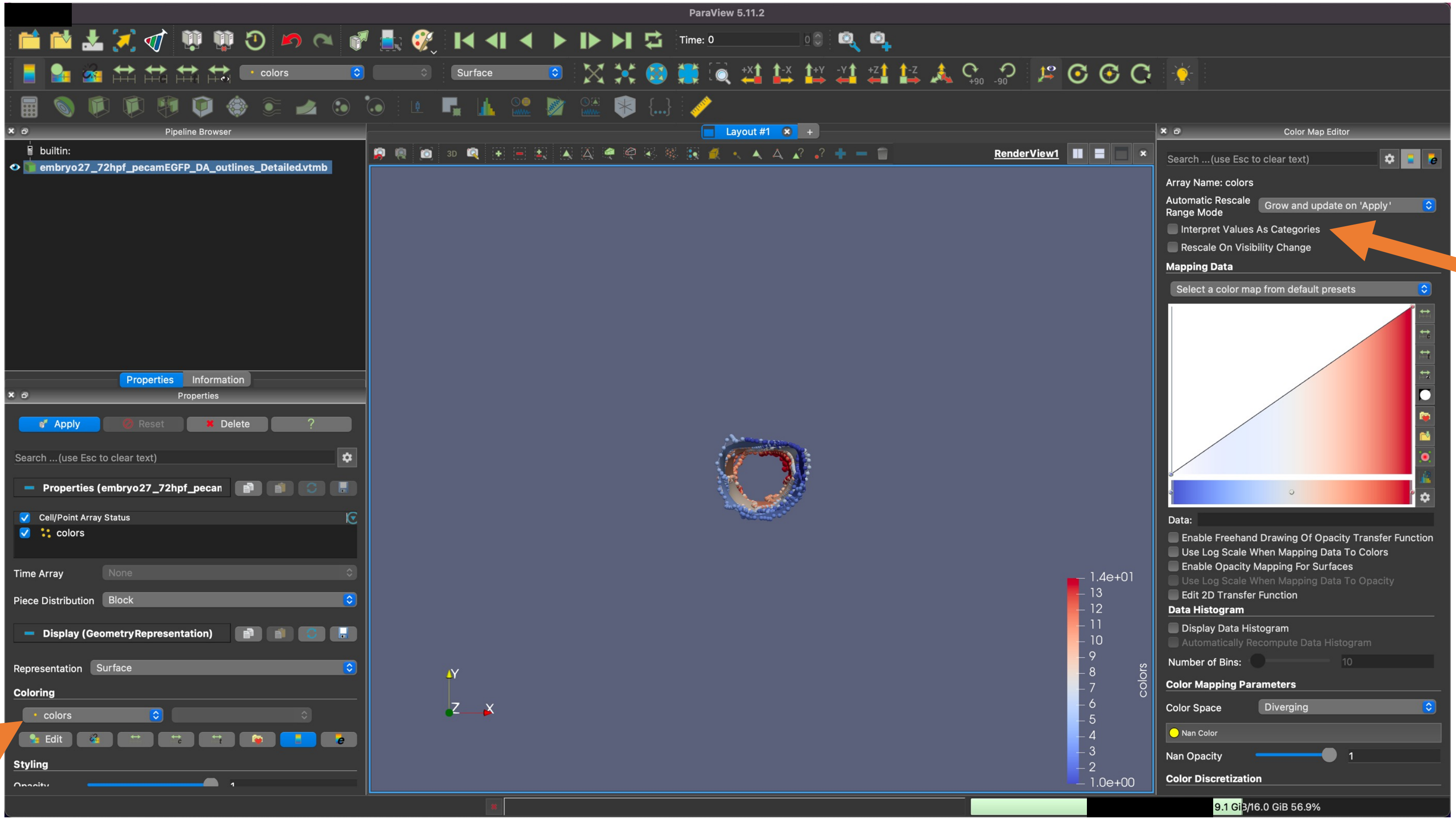

5) Tick 'Interpret Values as Categories'.

3) Choose 'colors'.
4) Click on 'Edit' below.

Coloring cells II

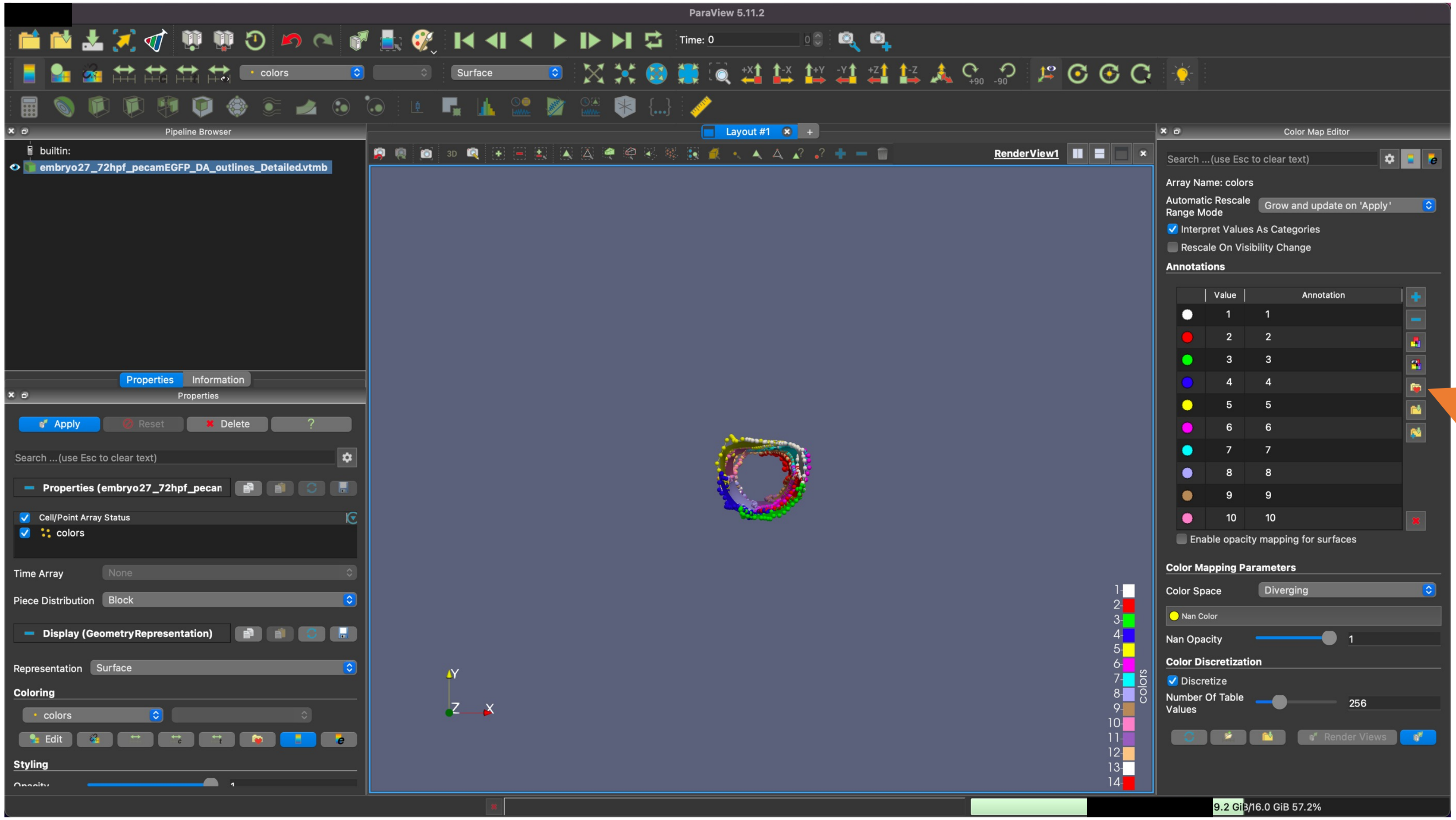

6) Click ‘Choose preset.’

Coloring cells III

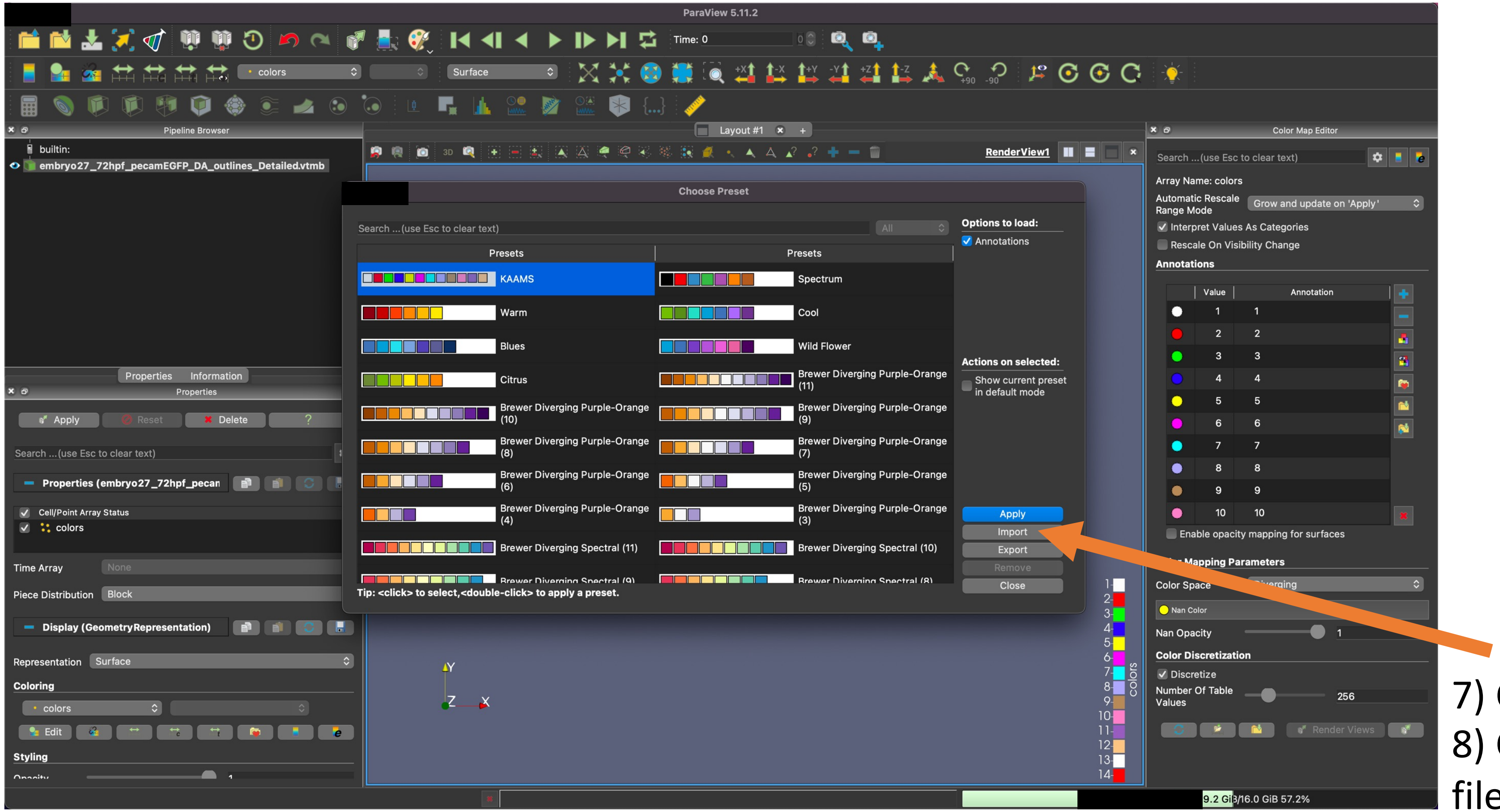

7) Click ‘Import’.
8) Choose provided file ’ec_colors.json’ *

* same colors as used in Fig 1 of the article

Coloring cells IV

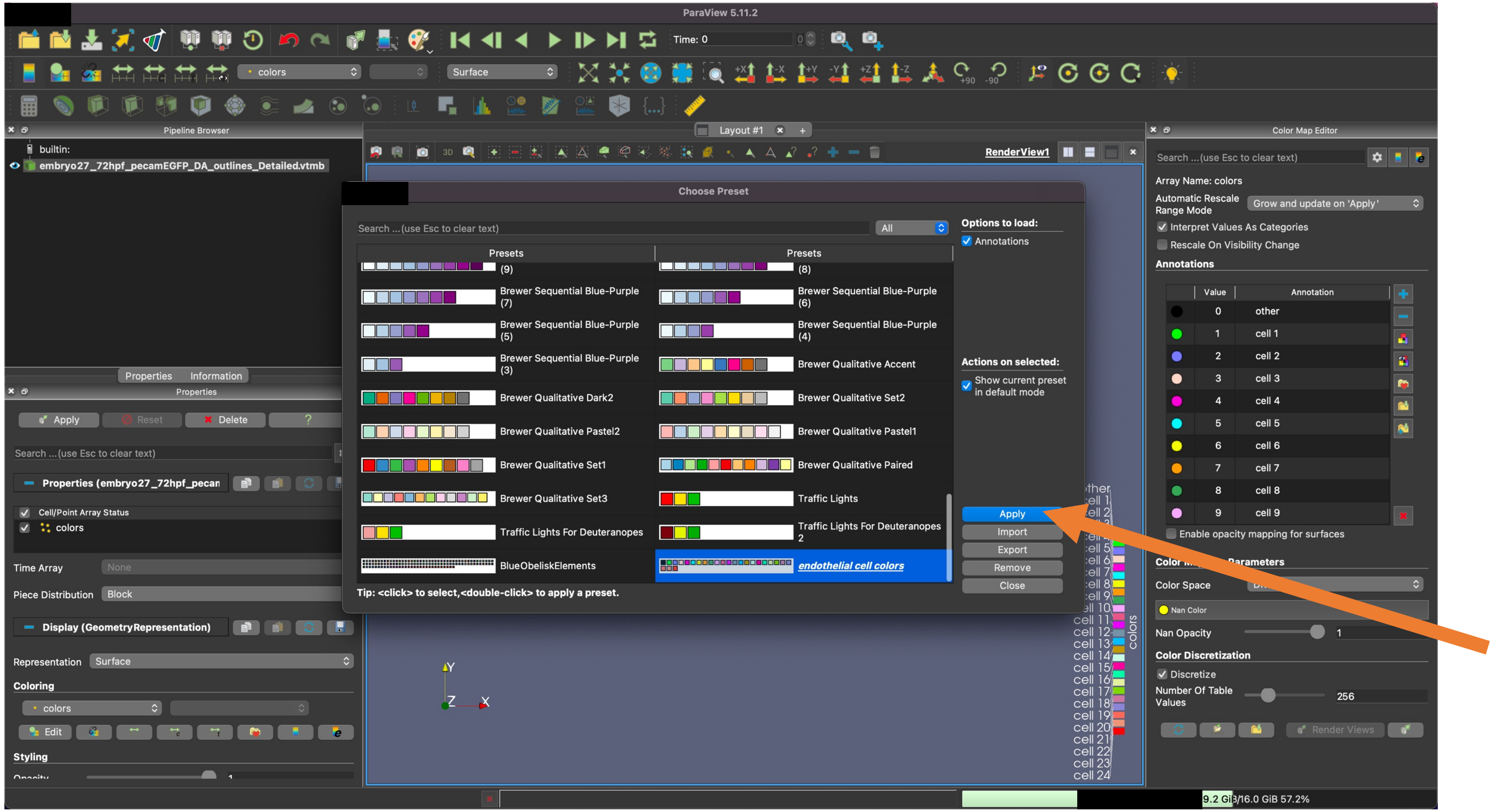

9) Click 'Apply'.
10) Click 'Close'.

Coloring cells V

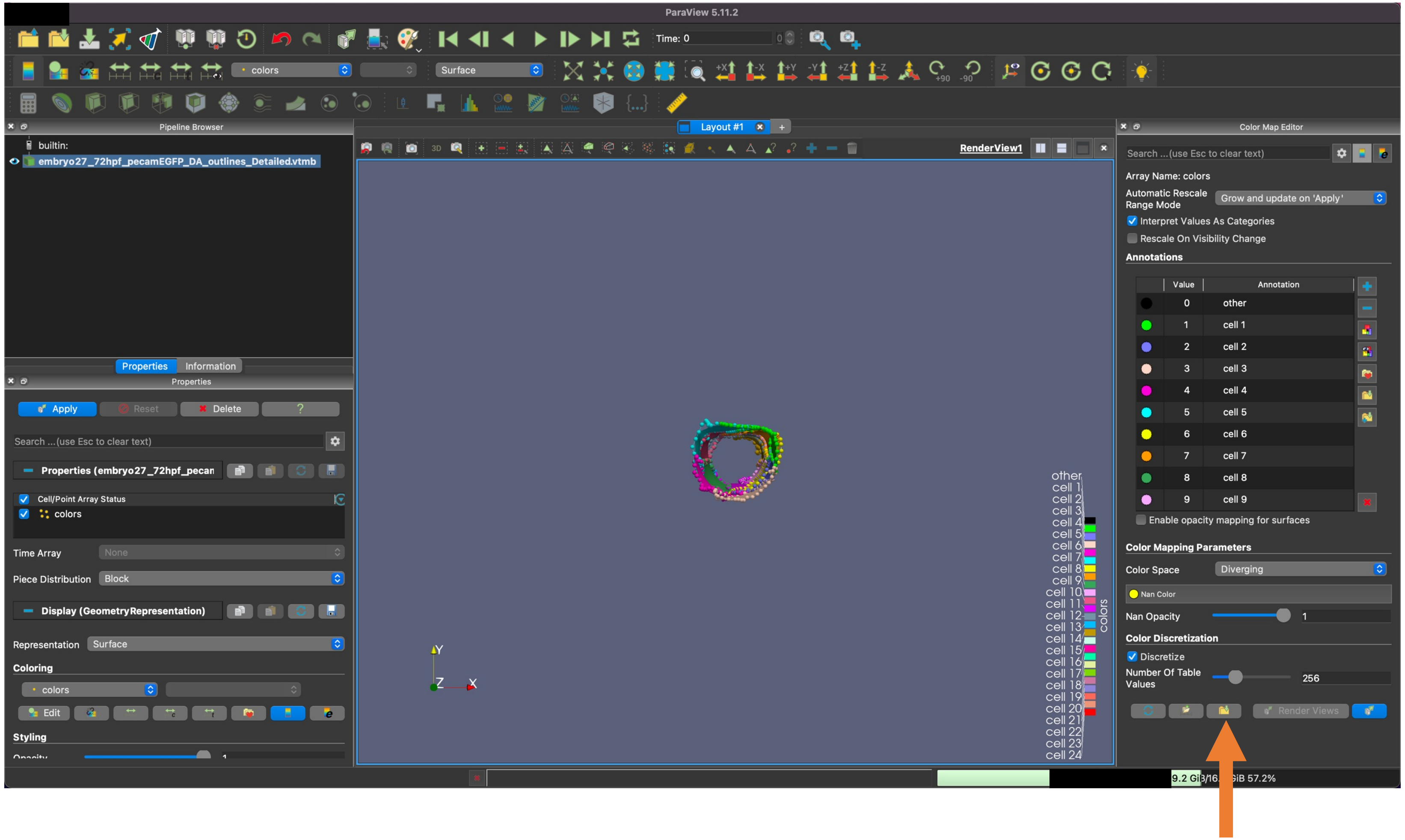

11) Click ‘Save current color map settings values as default for all arrays’.

Improve visibility of endothelial contours

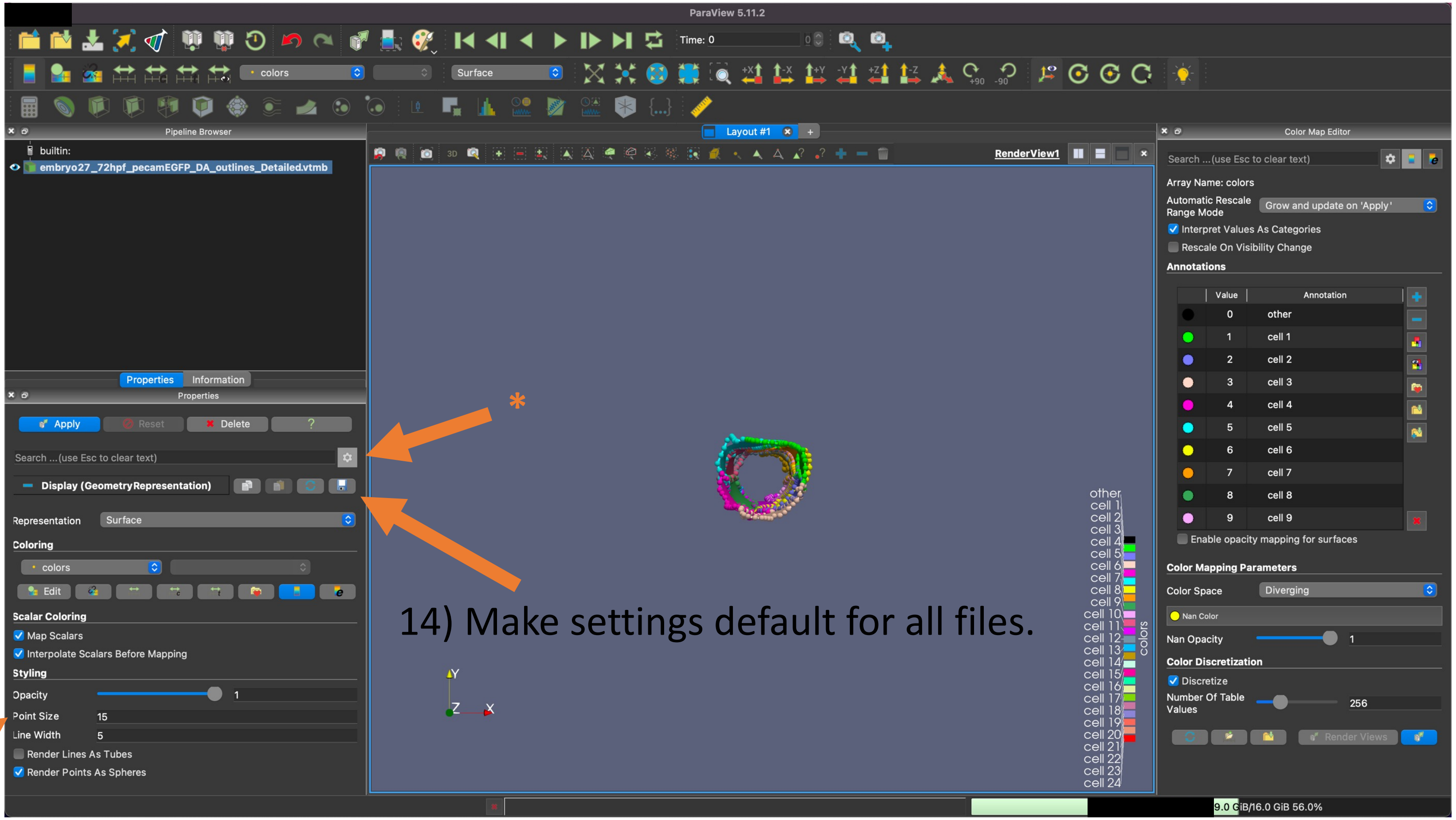

12) Increase ‘Point Size’ and ‘Line Width’ (click on *, if these options are invisible)
13) Tick ‘Render Points as Spheres’ below.

Lateral camera view

15) Click '+X'.

17) Click to save camera.

16) Click '+90'.

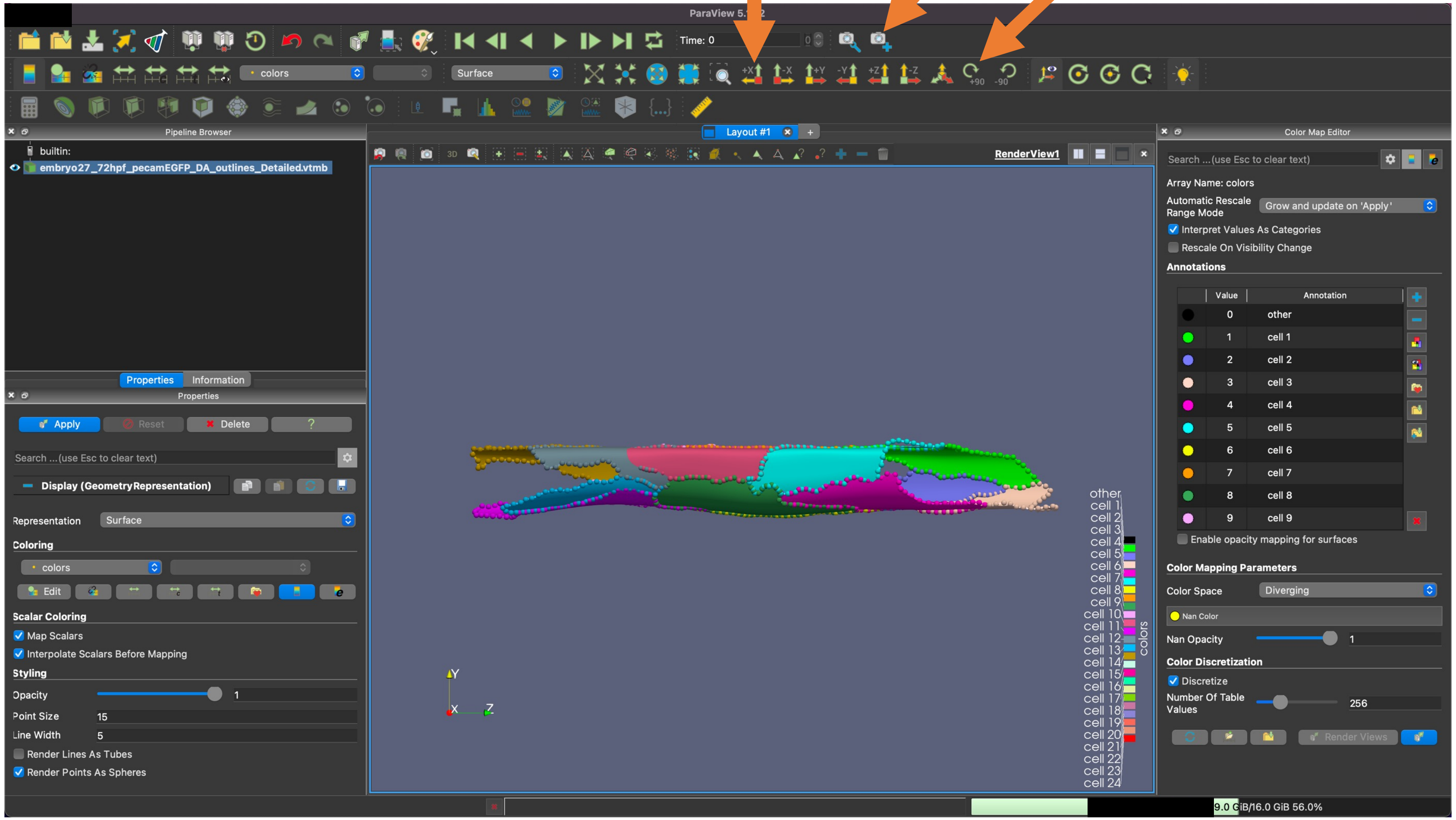

Transverse camera view

19) Click to save camera.

18) Click '-Z'.

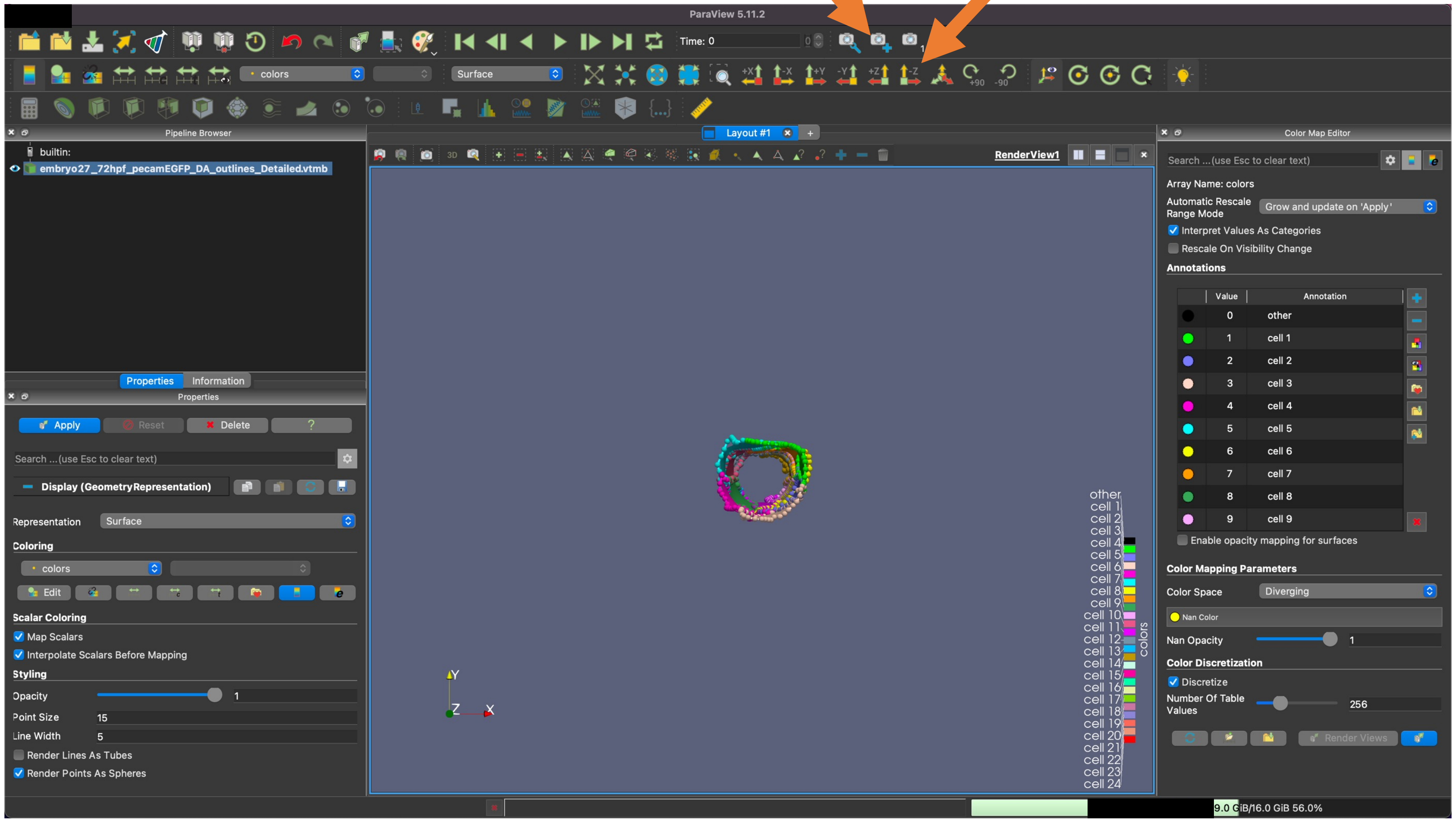

Improve smoothness of visualization

20) Open Preferences/Settings; click on ‘Render View’.

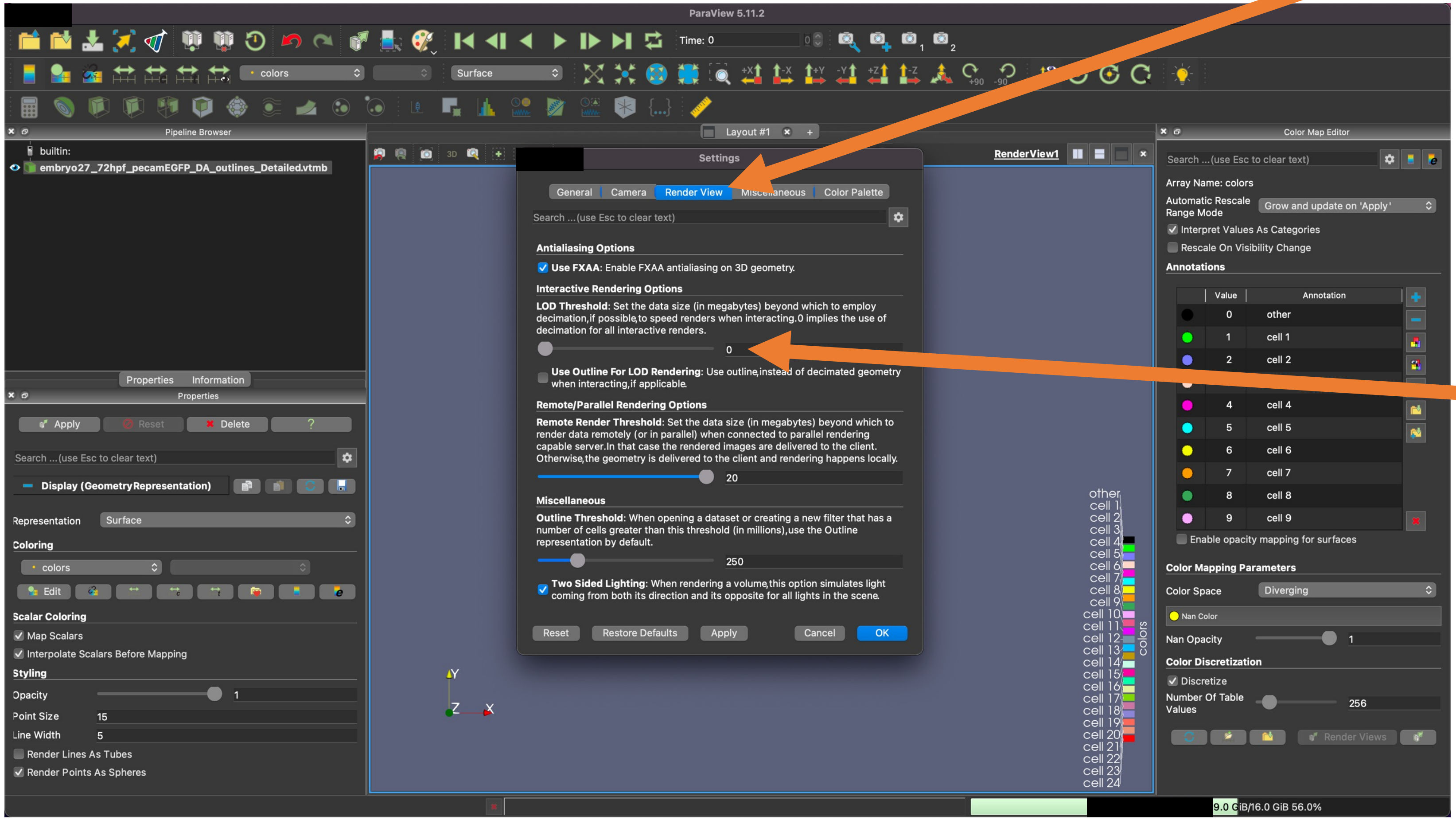

21) Increase this value to improve smoothness (uses more resources). Then click ‘Apply’.

Camera control

Further information, e.g., on how to move/rotate the camera *

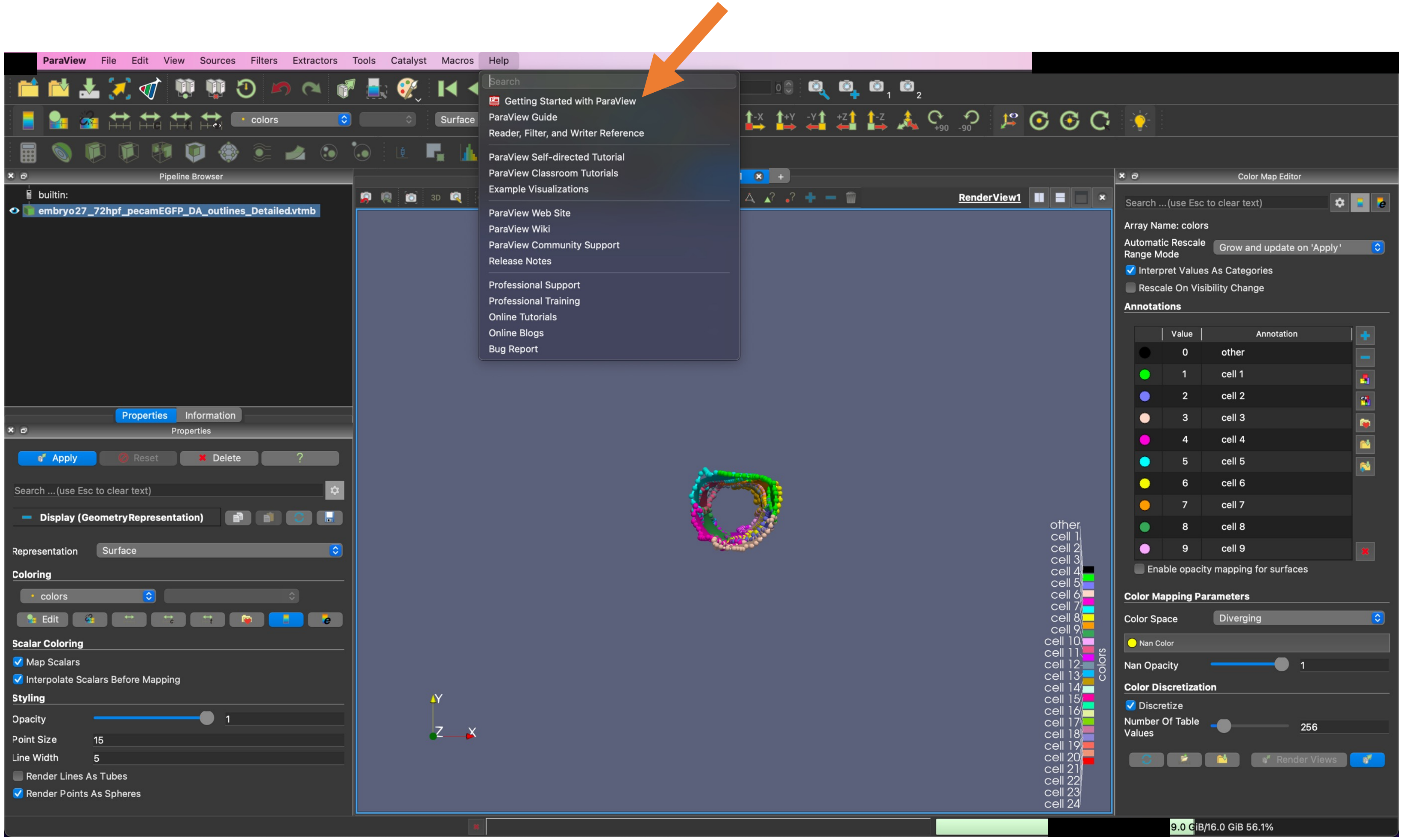

* For macOS:

Move:
Ctrl + shift + click & drag

Zoom:
Ctrl + click & drag

Rotate:
click & drag

Rotate in plane:
Opt + shift + click & drag

Viewing a new file

Close old file

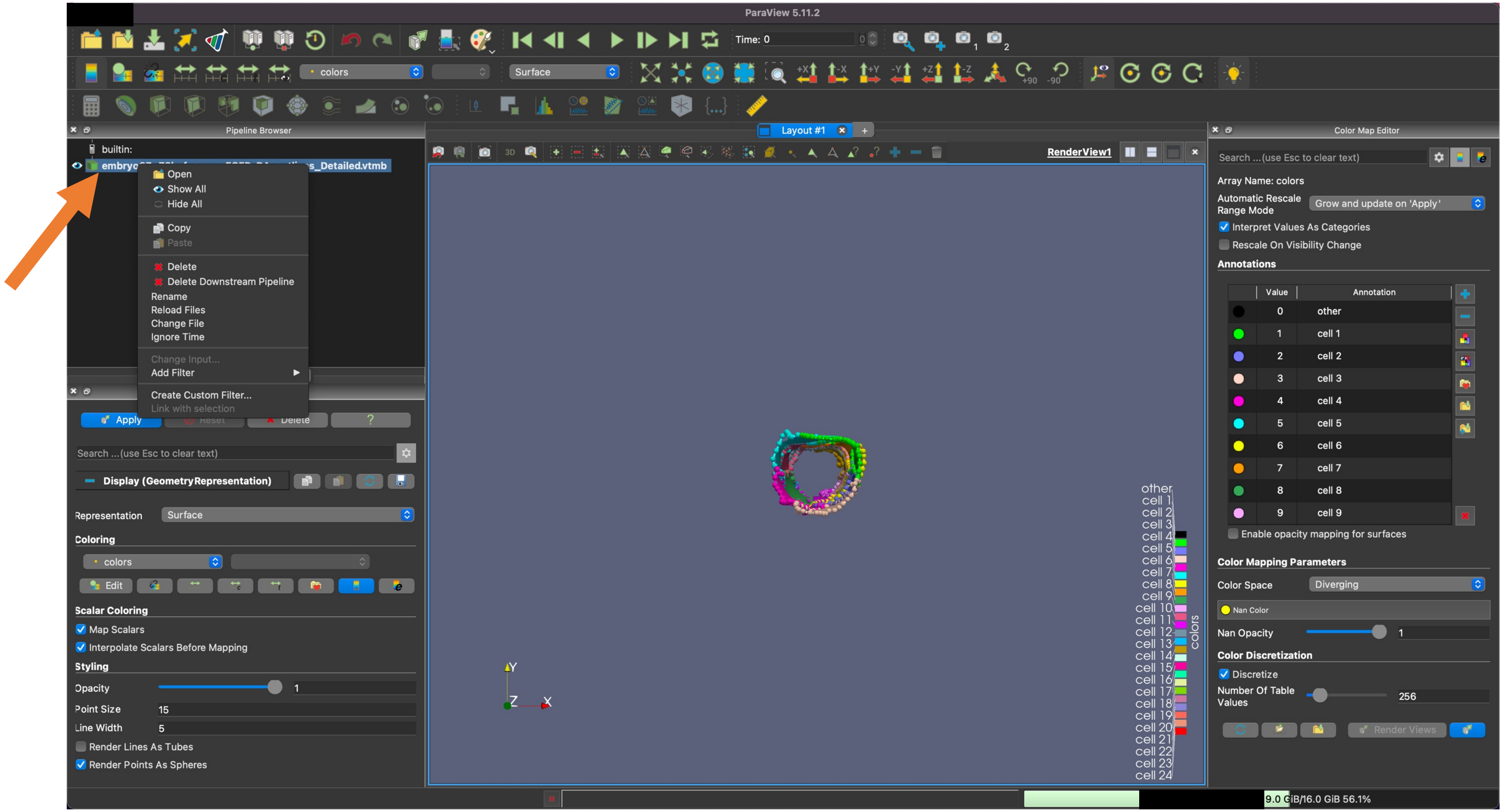

1) Right-click and choose 'Delete'.

Open new file

4) Restore camera settings.

2) Open new file.

3) Make visible.

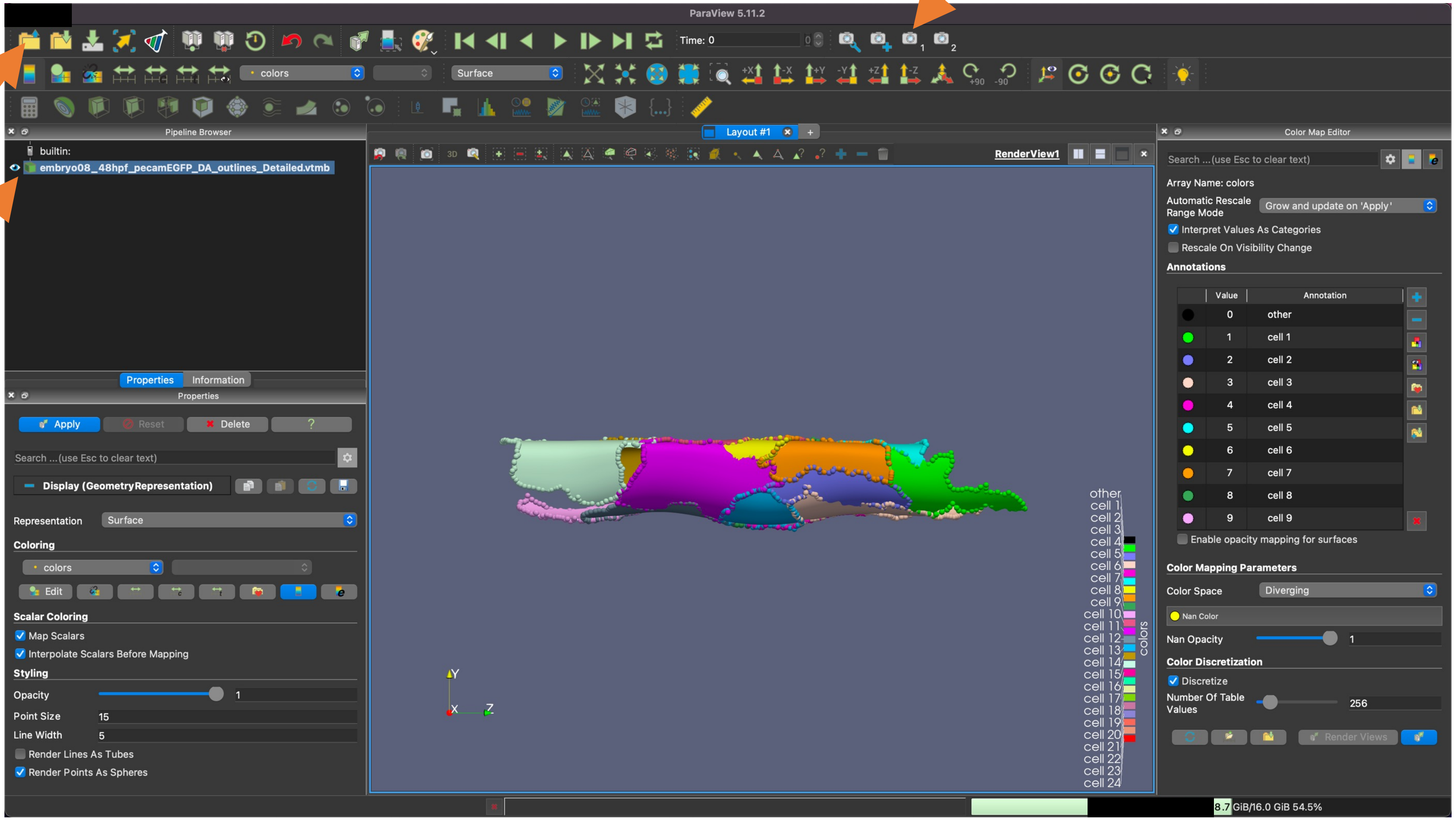

Supplement: S1 Tutorial — (PDF) [file pcbi.1011924.s002.pdf]
