# Supplementary material for: Novel mathematical approach to accurately quantify 3D endothelial cell morphology and vessel geometry based on fluorescently marked endothelial cell contours: Application to the dorsal aorta of wild-type and Endoglin-deficient zebrafish embryos
Source: PLoS Comput Biol. 2024 Aug 30;20(8):e1011924. doi: 10.1371/journal.pcbi.1011924 (PMC11392406; doi:10.1371/journal.pcbi.1011924)
Supplement: S1 Table — (PDF) [file pcbi.1011924.s020.pdf]

S1 Table. Frequently used mathematical symbols.

Context	Symbol	First mention	Description
cell contour	EC_ℓ	general notation	collection of cell contours of all N endothelial cells of the same type, i.e., $\ell \in \{\mathrm{anno}, \mathrm{enri}, \mathrm{spline}, \mathrm{transf}, \mathrm{proj}\}$
	$\mathrm{EC}_{\mathrm{anno},i}$	step 1a)	contour of endothelial cell i manually annotated in Imaris software
	$\mathrm{EC}_{\mathrm{enri},i}$	step 1b)	enrichment of contour $\mathrm{EC}_{\mathrm{anno},i}$ by information from neighboring cells and interpolation on contour edges
	$\mathrm{EC}_{\mathrm{spline},i}$	step 1c)	equidistant points computed on smoothing spline $s_i(u)$ that was fitted to contour $\mathrm{EC}_{\mathrm{enri},i}$
	$\mathrm{EC}_{\mathrm{transf},i}$	step 1d)	coordinate transformation of contour $\mathrm{EC}_{\mathrm{spline},i}$ from original coordinate system within microscopy image to coordinate system that was estimated from $\mathrm{EC}_{\mathrm{spline}}$
	$\mathrm{EC}_{\mathrm{proj},i}$	step 3a)	projection of spline $s_{\mathrm{transf},i}(u)$ onto cross-sectional shapes $\tilde{\varphi}_k$
cell contour spline	$s_i(u)$	step 1c)	smoothing spline fitted to $\mathrm{EC}_{\mathrm{enri},i}$
	$s_{\mathrm{transf},i}(u)$	step 1d)	coordinate transformation of spline s_i from original coordinate system within microscopy image to coordinate system that was estimated from $\mathrm{EC}_{\mathrm{spline}}$
cross-sectional shape	$\bar{\varphi}(u) := \varphi(u; \bar{\theta})$	step 1d)	mean cross-sectional shape with parameters $\bar{\theta}$ estimated from $\mathrm{EC}_{\mathrm{spline}}$
	$\varphi_k(u) := \varphi(u; \theta_k)$	step 2a)	cross-sectional shape with parameters θ_k estimated at position z_k along the DA's anterior-posterior (z-)axis from $\mathrm{EC}_{\mathrm{transf}}$
	$\tilde{\varphi}_k(u) := \varphi(u; \tilde{\theta}(z_k))$	step 2b)	cross-sectional shape at position z_k obtained by smoothing of the cross-sectional shapes φ_k along the DA's anterior-posterior axis
	CS_k	step 2c)	approximation of cross-sectional shape $\tilde{\varphi}_k$ by equidistant points
	CS	step 2c)	collection of equidistant points on all M cross-sectional shapes
point	p^+	general notation	(periodic) successor of p on endothelial cell contour or cross-sectional shape
	p^-		(periodic) predecessor of p on endothelial cell contour or cross-sectional shape
