# Supplementary material for: Novel mathematical approach to accurately quantify 3D endothelial cell morphology and vessel geometry based on fluorescently marked endothelial cell contours: Application to the dorsal aorta of wild-type and Endoglin-deficient zebrafish embryos
Source: PLoS Comput Biol. 2024 Aug 30;20(8):e1011924. doi: 10.1371/journal.pcbi.1011924 (PMC11392406; doi:10.1371/journal.pcbi.1011924)
Supplement: S2 Table — (PDF) [file pcbi.1011924.s021.pdf]

S2 Table. Identified tuning parameter values.

Appearance	Symbol	Function	Value
Step 1b) Enrichment of endothelial cell contours	r	radius of cylinders centered around edges of the contour that is enriched by information from neighboring cells	$0.65\,\mu m$
	Δh	length extension of cylinders centered around edges of the contour that is enriched by information from neighboring cells	$0.05\,\mu m$
	$n_{\text{interp,rel}}$	relative number of points linearly interpolated on cell contour edges	$1\,\mu m^{-1}$
Step 1c) Smoothing of endothelial cell contours	ϵ_{spline}	upper bound on the allowed mean squared distance of fitted contour splines to enriched cell contours	$(0.15\,\mu m)^2$
	$n_{\text{spline,rel}}$	relative number of equidistant points computed on cell contour splines	$4\,\mu m^{-1}$
Step 2a) Estimation of vessel cross-sections	M_{rel}	relative number of vessel cross-sections estimated along the anterior-posterior axis	$5\,\mu m^{-1}$
	n_{oct}	minimal number of points with non-zero weight per plane octant	30
	Z_ω	controls the distance of the truncation point in either direction from the mean value of the Gaussian function underlying the employed weight function	4
	λ	upper bound on the allowed relative deviation of the locally estimated cross-sectional shape from the mean shape	20 %
	n_{poly}	number of points for the polygonal approximation of the current estimate of the local cross-sectional shape	1000
Step 2b) Smoothing of vessel surface	σ	standard deviation of the Gaussian function underlying the employed smoothing function	$10\,\mu m$
	Z_σ	controls the distance of the truncation point in either direction from the mean value of the Gaussian function underlying the employed smoothing function	4
Step 2c) Computation of grid	$n_{\text{cross,rel}}$	relative number of points computed on each smoothed cross-sectional shape	$15\,\mu m^{-1}$
