# Supplementary material for: Novel mathematical approach to accurately quantify 3D endothelial cell morphology and vessel geometry based on fluorescently marked endothelial cell contours: Application to the dorsal aorta of wild-type and Endoglin-deficient zebrafish embryos
Source: PLoS Comput Biol. 2024 Aug 30;20(8):e1011924. doi: 10.1371/journal.pcbi.1011924 (PMC11392406; doi:10.1371/journal.pcbi.1011924)
Supplement: S3 Table — Reference vessel cross-sectional shapes estimated with noct = 30, λ = 20% and σ = 10 μm. Each vessel cross-sectional shape re-estimated after altering either noct, λ or σ, while fixing the other two parameters. Relative deviations devcross of the re-estimated vessel cross-sectional shapes from the reference shapes computed with Eq (16) (main text); results for pooled vessel cross-sectional shapes from wild-type and Endoglin-deficient embryos at 48 hpf and 72 hpf. When noct = 60, the number of cross-sections was 24 265. In all other computations, the number of cross-sections was 25 111. †: constraint from Eq (18) (main text) omitted. *: vessel surface not smoothed. When halving or doubling either noct, λ or σ, the majority of estimated vessel cross-sections had a relative deviation of less than 5%. Notably, the largest median differences were found when using the mean shape only (λ = 0%), followed by the setting where smoothing was omitted. The largest maximal differences were obtained when not constraining the deviation of the local cross-sectional shape from the mean shape. (PDF) [file pcbi.1011924.s022.pdf]

S3 Table. Robustness of chosen tuning parameter values w.r.t. cross-sectional geometry.

n_{oct}	λ in %	σ in µm	Median (Min, Max) of dev_{cross} in %
1	20	10	0.8 (0.0, 11)
15			0.4 (0.0, 12)
60			0.9 (0.0, 23)
30	0	10	12 (3.4, 24)
	10		4.7 (0.0, 15)
	40		0.8 (0.0, 26)
	NA†		0.8 (0.0, 83)
30	20	NA*	5.9 (0.6, 21)
		5	3.1 (0.3, 8.5)
		20	4.5 (0.9, 11)
