# Supplementary material for: Novel mathematical approach to accurately quantify 3D endothelial cell morphology and vessel geometry based on fluorescently marked endothelial cell contours: Application to the dorsal aorta of wild-type and Endoglin-deficient zebrafish embryos
Source: PLoS Comput Biol. 2024 Aug 30;20(8):e1011924. doi: 10.1371/journal.pcbi.1011924 (PMC11392406; doi:10.1371/journal.pcbi.1011924)
Supplement: S4 Table — Reference cell morphometric measurements computed after vessel surface reconstruction with noct = 30, λ = 20% and σ = 10 μm. Morphology of each cell re-computed after altering either noct, λ or σ, while fixing the other two parameters. Relative deviations devrel of re-computed cell morphometric measurements from their reference values computed with Eq (37) (main text); results for pooled EC surfaces from wild-type and Endoglin-deficient embryos at 48 hpf and 72 hpf. f: relative frequency. Note: We omitted the column f(devrel < −10%) as it can be computed as f(devrel < −10%) = 100% − f(|devrel| ≤ 10%) − f(devrel > 10%). When noct = 60, the number of cells was 290. In all other computations, the number of cells was 296. †: constraint from Eq (18) (main text) omitted. *: vessel surface not smoothed. When halving or doubling either noct, λ or σ, each morphometric measurement differed by maximally 10%. Notably, when not smoothing, larger cell surface areas were computed. As a consequence, elongation was decreased and compactness increased. (PDF) [file pcbi.1011924.s023.pdf]

S4 Table. Robustness of chosen tuning parameter values w.r.t. endothelial cell morphology.

Measure	n_{oct}	λ in %	σ in µm	$f(\lvert dev_{rel}\rvert \leq 10\,\%)$ in %	$f(dev_{rel} > 10\,\%)$ in %
Area	1	20	10	100	0.0
	15			100	0.0
	60			100	0.0
	30	0	10	96	2.4
		10		100	0.0
		40		100	0.0
		NA†		99.7	0.3
	30	20	NA*	86	14
			5	100	0.0
			20	100	0.0
Perimeter	1	20	10	100	0.0
	15			100	0.0
	60			100	0.0
	30	0	10	100	0.0
		10		100	0.0
		40		100	0.0
		NA†		100	0.0
	30	20	NA*	100	0.0
			5	100	0.0
			20	100	0.0
Compactness	1	20	10	100	0.0
	15			100	0.0
	60			100	0.0
	30	0	10	99.3	0.7
		10		100	0.0
		40		100	0.0
		NA†		100	0.0
	30	20	NA*	91	9.5
			5	100	0.0
			20	100	0.0
Elongation	1	20	10	100	0.0
	15			100	0.0
	60			100	0.0
	30	0	10	97	1.4
		10		100	0.0
		40		100	0.0
		NA†		100	0.0
	30	20	NA*	91	0.0
			5	100	0.0
			20	100	0.0
