# Supplementary material for: Novel mathematical approach to accurately quantify 3D endothelial cell morphology and vessel geometry based on fluorescently marked endothelial cell contours: Application to the dorsal aorta of wild-type and Endoglin-deficient zebrafish embryos
Source: PLoS Comput Biol. 2024 Aug 30;20(8):e1011924. doi: 10.1371/journal.pcbi.1011924 (PMC11392406; doi:10.1371/journal.pcbi.1011924)
Supplement: S5 Table — Relative deviations devrel of the luminal areas of slices of the estimated vessel surfaces from angiogram slices for two wild-type embryos at 72 hpf with two sets of cell contour annotations each (validation data), computed with Eq (37) (main text). Vessel cross-sections estimated from either of the two versions of annotated cell contours pooled per embryo. f: relative frequency. Note: We omitted the column f(devrel < −10%) as it can be computed as f(devrel < −10%) = 100% − f(|devrel| ≤ 10%) − f(devrel > 10%). The number of vessel surface slices was 90 for embryo 14 and 102 for embryo 15. The superellipse performed best for embryo 14 and the ellipse performed best for embryo 15. In embryo 14, more luminal areas were under- than overestimated, in embryo 15 vice versa. (PDF) [file pcbi.1011924.s024.pdf]

S5 Table. Compliance of luminal areas of estimated cross-sectional shapes with angiography.

Embryo	Shape	$f(\lvert \mathrm{dev_{rel}} \rvert \leq 10\,\%)$ in %	$f(\mathrm{dev_{rel}} > 10\,\%)$ in %	$f(\lvert \mathrm{dev_{rel}} \rvert \leq 20\,\%)$ in %	$f(\mathrm{dev_{rel}} > 20\,\%)$ in %
14	circle	54	13	94	1.1
	ellipse	64	7.8	97	1.1
	superellipse	84	4.4	100	0.0
15	circle	47	38	90	9.8
	ellipse	62	36	87	13
	superellipse	46	48	87	13
