# Supplementary material for: Novel mathematical approach to accurately quantify 3D endothelial cell morphology and vessel geometry based on fluorescently marked endothelial cell contours: Application to the dorsal aorta of wild-type and Endoglin-deficient zebrafish embryos
Source: PLoS Comput Biol. 2024 Aug 30;20(8):e1011924. doi: 10.1371/journal.pcbi.1011924 (PMC11392406; doi:10.1371/journal.pcbi.1011924)
Supplement: S6 Table — Analysis based on cross-sections with at least two annotated cells present within a distance of 0.5 μm to the cross-sectional plane. The number of vessel cross-sections was 5575/5972 for wild-type embryos and 4682/5185 for Endoglin-deficient embryos at 48 hpf/72 hpf, respectively. *: individual measurements weighted by numbers of cross-sectional shapes per embryo (see Section 12 of S1 Appendix). Literature values digitized from graphics using WebPlotDigitizer; except for [33]: values found in text. [38]: Mean and s.d. in S2(J) Fig obscured by data points and could not be digitized. ¤: absolute relative deviation of literature mean/median value from our measured mean/median value is greater than 20%. s.d.: standard deviation. s.e.m.: standard error of mean. Q1: 25% percentile. Q2: median. Q3: 75% percentile. (PDF) [file pcbi.1011924.s025.pdf]

S6 Table. Comparison to literature-reported geometric measurements of dorsal aorta ***(continued on next page).***

Measure	Phenotype	Time	Mean ± s.d.* (Min, Max)	Literature Value	Literature Type	Literature Source
luminal diameter in µm	wt	48 hpf	28.9 ± 2.62 (21.4, 33.6)	32.3	single value	Fig 3J [32]
				22.6 ± 2.54¤	mean ± s.d.	S1A Fig [34]
				27.0 ± 0.452	mean ± s.e.m.	Fig 3A [3]
				22.3 (18.9, 24.0)¤	Q_2 (Q_1, Q_3)	Fig 4B [36]
				21.5 (18.5, 23.6)¤	Q_2 (Q_1, Q_3)	Fig 4B [36]
				22.0 (19.3, 24.0)¤	Q_2 (Q_1, Q_3)	Fig 4B [36]
				20.7 (18.0, 23.3)¤	Q_2 (Q_1, Q_3)	Fig 4B [36]
				21.4 ± 3.14¤	mean ± s.d.	Fig 1O [38]
				19.6 ± 2.06¤	mean ± s.d.	Fig 4G [38]
				27.3 ± 3.62	mean ± s.d.	Fig 7R [39]
		53 hpf		22.7 ± 2.93	mean ± s.d.	Fig 2K [39]
		72 hpf	18.0 ± 1.46 (15.4, 21.9)	17.2	single value	Fig 3J [32]
				29.6 ± 0.77¤	mean ± s.e.m.	Fig 3C [33]
				19.8 ± 3.40	mean ± s.d.	S1A Fig [34]
				21.0 ± 0.415	mean ± s.e.m.	Fig 2F [3]
				21.0 ± 0.422	mean ± s.e.m.	Fig 3A [3]
				13.5 ± 1.41¤	mean ± s.d.	Fig 7A2 [35]
				18.4 (17.1, 21.0)	Q_2 (Q_1, Q_3)	Fig 4B [36]
				18.4 (17.1, 20.8)	Q_2 (Q_1, Q_3)	Fig 4B [36]
				18.0 (16.7, 20.0)	Q_2 (Q_1, Q_3)	Fig 4B [36]
				17.7 (16.5, 18.5)	Q_2 (Q_1, Q_3)	Fig 4B [36]
				20.8 ± 2.25	mean ± s.d.	Fig 3D [37]
				20.2 ± 2.58	mean ± s.d.	Fig 4C [37]
				20.9 ± 2.47	mean ± s.d.	Fig 4E [37]
				20.1 ± 1.57	mean ± s.d.	Fig 5A [37]
				20.4 ± 2.28	mean ± s.d.	S6A Fig [37]
				20.6 ± 1.66	mean ± s.d.	S6C Fig [37]
				21.6 ± 1.85	mean ± s.d.	Fig 2K [39]
				15.1 ± 0.644	mean ± s.e.m.	Fig 4C [40]
				15.0 ± 1.14	mean ± s.e.m.	Fig 4C [40]
				15.2 ± 0.590	mean ± s.e.m.	Fig 5E [40]
				15.0 ± 1.21	mean ± s.e.m.	Fig 5E [40]
				13.1 ± 2.73¤	mean ± s.e.m.	S2C Fig [40]
				13.4 ± 1.16¤	mean ± s.e.m.	S2C Fig [40]

S6 Table. Comparison to literature-reported geometric measurements of dorsal aorta *(continued).*

**Measure**	**Phenotype**	**Time**	**Mean ± s.d.*** **(Min, Max)**	**Literature**		
				Value	Type	Source
luminal diameter in µm	Eng-def	48 hpf	24.2 ± 2.26 (19.4, 29.3)	26.3 ± 0.695	mean ± s.e.m.	Fig 3C [3]
		72 hpf	27.6 ± 2.55 (23.6, 33.3)	27.7 ± 0.491	mean ± s.e.m.	Fig 2F [3]
				27.7 ± 0.514	mean ± s.e.m.	Fig 3C [3]
				27.7 ± 0.468	mean ± s.e.m.	S4D Fig [3]
				26.7 ± 1.48	mean ± s.d.	Fig 3D [37]
				26.8 ± 2.31	mean ± s.d.	Fig 4C [37]
				27.1 ± 1.20	mean ± s.d.	Fig 4E [37]
				26.7 ± 1.03	mean ± s.d.	Fig 5A [37]
				27.0 ± 2.40	mean ± s.d.	S6A Fig [37]
				27.4 ± 1.24	mean ± s.d.	S6C Fig [37]
luminal area in μm^2	wt	48 hpf	590 ± 98.3 (330, 798)	585 ± 64.7	mean ± s.d.	Fig 1D [16]
				624 ± 50.4	mean ± s.d.	Fig 2B [16]
				613 ± 77.0	mean ± s.d.	Fig 4A [16]
				772 ± 181[¤]	mean ± s.d.	S2D Fig [16]
				498 ± 153	mean ± s.d.	S3B Fig [16]
				607 ± 70.8	mean ± s.d.	S4E Fig [16]
		72 hpf	217 ± 30.7 (154, 283)	229 ± 33.4	mean ± s.d.	Fig 1D [16]
