# Supplementary material for: Novel mathematical approach to accurately quantify 3D endothelial cell morphology and vessel geometry based on fluorescently marked endothelial cell contours: Application to the dorsal aorta of wild-type and Endoglin-deficient zebrafish embryos
Source: PLoS Comput Biol. 2024 Aug 30;20(8):e1011924. doi: 10.1371/journal.pcbi.1011924 (PMC11392406; doi:10.1371/journal.pcbi.1011924)
Supplement: S7 Table — Analysis based on ECs with projection distances (onto the estimated vessel surfaces) comparable to or lower than the maximal annotation uncertainty of 0.742 μm. The number of cells was 73/62 for wild-type embryos and 71/68 for Endoglin-deficient embryos at 48 hpf/72 hpf, respectively. *: individual measurements weighted by numbers of cells per embryo (see Section 12 of S1 Appendix). Literature values digitized from graphics using WebPlotDigitizer. [3]: compactness not computed; definition of elongation differed from ours. ¤: absolute relative deviation of literature mean value from our measured mean value is greater than 20%. s.d.: standard deviation. s.e.m.: standard error of mean. EHT: ECs undergoing endothelial-to-hematopoietic transition. EHT?: potentially hemogenic cells. (PDF) [file pcbi.1011924.s026.pdf]

S7 Table. Comparison to literature-reported morphological measurements of endothelial cells.

Measure	Phenotype	Time	Mean ± s.d.* (Min, Max)	Literature		
				Value	Type	Source
surface area in µm^2	wt	48 hpf	846 ± 299 (314, 2280)	943 ± 64.5	mean ± s.e.m.	Fig 7F [3]
				1230 ± 82.4¤	mean ± s.e.m.	Fig 4D (EC) [14]
				716 ± 112	mean ± s.e.m.	Fig 4D (EHT?) [14]
				174 ± 22.5¤	mean ± s.e.m.	Fig 4D (EHT) [14]
				587 ± 79.1¤	mean ± s.d.	Fig 1D [16]
				808 ± 97.0	mean ± s.d.	Fig 2B [16]
				669 ± 125¤	mean ± s.d.	Fig 4A [16]
				570 ± 103¤	mean ± s.d.	S3B Fig [16]
				827 ± 108	mean ± s.d.	S4E Fig [16]
				256 ± 75.1¤	mean ± s.d.	Fig 8K [38]
				281 ± 86.4¤	mean ± s.d.	Fig 8L [38]
				214 ± 53.8¤	mean ± s.d.	S11C Fig [38]
		72 hpf	781 ± 294 (394, 2240)	1030 ± 54.1¤	mean ± s.e.m.	Fig 7F [3]
				819 ± 264	mean ± s.d.	Fig 1D [16]
	Eng-def	48 hpf	662 ± 283 (189, 1720)	743 ± 41.3	mean ± s.e.m.	Fig 7F [3]
		72 hpf	979 ± 404 (231, 2710)	1270 ± 63.5¤	mean ± s.e.m.	Fig 7F [3]
perimeter in µm	wt	48 hpf	143 ± 31.5 (67.2, 242)	157 ± 7.86	mean ± s.e.m.	Fig 7G [3]
		72 hpf	157 ± 24.7 (113, 225)	177 ± 7.14	mean ± s.e.m.	Fig 7G [3]
	Eng-def	48 hpf	126 ± 31 (64.6, 219)	144 ± 6.97	mean ± s.e.m.	Fig 7G [3]
		72 hpf	170 ± 38.8 (67.8, 266)	203 ± 6.93	mean ± s.e.m.	Fig 7G [3]
