# Supplementary material for: Novel mathematical approach to accurately quantify 3D endothelial cell morphology and vessel geometry based on fluorescently marked endothelial cell contours: Application to the dorsal aorta of wild-type and Endoglin-deficient zebrafish embryos
Source: PLoS Comput Biol. 2024 Aug 30;20(8):e1011924. doi: 10.1371/journal.pcbi.1011924 (PMC11392406; doi:10.1371/journal.pcbi.1011924)
Supplement: Attachment — Submitted filename: comments2reviewers.pdf [file pcbi.1011924.s027.pdf]

Response to the reviewers' comments

Preface

We highly appreciated the constructive comments and remarks by the reviewers, as well as the opportunity to revise our manuscript. In the point-to-point response below, we have addressed all comments and remarks and revised the manuscript accordingly (with changes highlighted in italic).

Finally, we would like to thank the reviewers for their time and consideration.

Reviewer #1:

The work by Seeler et al. uses a newly developed mathematical morphology model to describe endothelial cell shapes within the dorsal aorta of wildtype and endoglin mutant zebrafish embryos. Their model accounts for the dorsal flattening of the dorsal aorta, while previous models assumed the vessel to be a cylinder. The authors also provide a nice visualization tool using paraview. Overall, the data seem to be of great quality and will better enable the analysis of endothelial cell shapes in the future.

> We appreciate the positive feedback on the manuscript.

This said, I was not able to judge or check the mathematics behind their calculations, due to my lack of expertise. However, regarding the biology of the system, I have several specific comments.

1. The authors claim that they have "identified a previously unrecognized dorsal-ventral asymmetry of EC morphology", as also the title states. However, the authors leave it undiscussed to what extent the smaller ventral cells they observe are hemogenic endothelial cells that are well described in zebrafish (e.g. Kissa and Herbomel, Blood and Nature papers). It is also established that they have a different morphology and are smaller (Lancino et al., elife 2018). The authors state that they excluded "protruding" cells from the DA (hematopoietic stem cells), but they leave it unclear, whether the smaller cells are on their way to become hematopoietic stem cells. Clearly some work on the biology would be necessary here. Do the smaller cells express runx1 or c-myb? Can the authors use transgenic zebrafish to monitor e.g. runx1 expression and then determine cell sizes to see whether smaller cells express runx1?

> We thank the reviewer for drawing our attention to ambiguities regarding our reasoning and the criterion for the exclusion of (potentially) hemogenic cells in our study. The goal of our study was to improve our understanding of the relationship between vessel geometry and endothelial cell (EC) morphology during vascular remodeling of the zebrafish dorsal aorta (DA). For this, we consider it important to analyze the morphology of all cells that actively contribute to DA geometry over a substantial time span. Our reasoning for the inclusion or exclusion of cells was therefore based on the cells' long-term impact on DA geometry and not on their genetic identity. Endothelial cells undergoing endothelial-to-hematopoietic transition (EHT) quickly extrude from the DA and thus do not contribute to vessel geometry over a substantial time span [1, 2]. As ECs in advanced stages of EHT can visually be discerned as cup-shaped blebs in the ventral sector of the DA [2], we deem a morphology-based criterion for the exclusion of these cells sufficient. In detail, we excluded cells during manual cell contour annotation if they were located at the ventral-most position of the DA and their contours, i.e., their cell junctions, were perfectly round and only circumscribed a small area (indicating blebbing). We now describe this criterion in more detail in the methods section.

We however acknowledge that without analyzing the expression of EC or hematopoietic stem and progenitor cell (HSPC) markers on individual cells, we cannot exclude the possibility that we excluded (small) ECs from our analysis or included cells in early stages of EHT in our analysis. This could have impacted our quantification of a dorsal-ventral asymmetry of EC morphology in the DA at 48 hpf. At 72 hpf however, the distinction between ECs and (precursor) HSPCs would not impact our analysis as EHT events reportedly stop by 60 hpf [1]. Our article now includes a discussion of our exclusion criterion.

We made the following change to the Materials and methods section:

Lines 177–181 in the clean manuscript:
During outlining, we excluded cells if they were located at the ventral most position of the DA and their contours, i.e., their cell junctions, were perfectly round and only circumscribed a small area. As these cells are likely (precursor) hematopoietic stem and progenitor stem cells (HSPCs) and would shortly extrude from the DA [2], they do not contribute to vessel geometry over a substantial time span.

We added the following paragraph to the discussion:

Lines 926–934 in the clean manuscript:
Lastly, we would like to note that our decision to exclude small round ventral cells from the analysis without staining for HSPC markers could have impacted our quantification of a dorsal-ventral asymmetry of EC morphology at 48 hpf*. The cells analyzed in this article could include larger (precursor) HSPCs or the excluded cells could include small endothelial cells. Future studies into the spatial organization of the early developing DA should combine morphometric measurements in dorsal and ventral cells with staining for HSPC markers. As the authors in [1] however detected no EHT events after* 60 hpf*, our quantification of a dorsal-ventral asymmetry at* 72 hpf *should not be affected by our exclusion of these small ventral ECs.*

2. How about statistical analysis? Are the size and other differences the authors measure statistically significant? Can the authors add this to the figures and legends?

Assessing statistical significance is crucial in hypothesis-driven research. Before performing hypothesis-driven research using a novel (mathematical) approach, however, it is important to thoroughly develop and evaluate the approach based on experimental data (see Fig S11 and Table S5–S7).

Following evaluation of our novel methodological approach, we performed an *exploratory* data analysis of vessel geometry and EC morphology in the DA of wild-type and Endoglin-deficient zebrafish embryos. Since we did not formulate hypotheses a priori regarding, e.g., the differences between dorsal and ventral cells, we did not test for statistical significance. Instead, we decided to fully explore the relation between endothelial cell surface morphology and cross-sectional geometry in the DA. We have added clarifications to the manuscript to emphasize that the presented research is exploratory and not hypothesis-driven.

Finally, we would like to point out that we quantified relative differences in medians for each comparison reported in the article. Hereby, we enable readers of our article to evaluate themselves whether for their exact question/hypothesis the reported morphological differences are *biologically* or *physiologically* significant. An example of this can be found in lines 726–728 in the clean manuscript:

"In wild-type embryos, we found evidence of a dorsal-ventral asymmetry. Firstly, dorsal ECs were larger than ventral ECs: Median surface area of dorsal ECs was 32 %/22 % larger at 48 hpf/72 hpf, respectively (see Fig 11A)."

We made the following changes to the abstract:
By simultaneously reconstructing vessel cross-sections and cell surfaces, we found in an exploratory analysis that morphology varied between endothelial cells located in different sectors of the dorsal aorta in both wild-type and Endoglin-deficient zebrafish embryos:

We made the following changes to the author summary:
In an exploratory analysis, we applied this method to wild-type and mutant zebrafish embryos lacking functional Endoglin that is required for the physiological vessel diameter decrease.

We added the following sentence to the Results section:

Lines 628–630 of the clean manuscript:
Subsequently, we applied our novel mathematical approach for an exploratory analysis of DA geometry and EC morphology in wild-type and Endoglin-deficient zebrafish embryos.

3. Are there different EC cell populations (e.g. based on size) in the ventral DA? An examination of the data the authors provide shows that often larger cells enwrap one side of the DA and greatly extend into the ventral area of the DA, while cells that are only located ventrally indeed appear smaller. How do the authors account for this morphology in their analysis?

We thank the reviewer for this suggestion of a more detailed analysis of spatial differences in EC morphology. In our article's analysis, we classified a cell as ventral if more than 50 % of its surface area was located in the ventral sector. This meant that (i) cells that were only located in the ventral sector, and (ii) cells that had most of their area in the ventral sector but also wrapped around the side of the DA were both treated as ventral ECs in our analysis.

We now added a follow-up analysis where we distinguished these two groups of cells. Using this finer classification, we found that the vast majority of the analyzed dorsal cells wrapped around the side of the DA, while a large group of ventral cells was exclusively located in the ventral sector. Morphological differences between these two subpopulations were in line with our finding of a dorsal-ventral asymmetry of EC morphology.

We added the following sentence to the Results section:

Lines 790–792 of the clean manuscript:
We confirmed these findings using a finer classification (see Section 9.5 in S1 Appendix), where we distinguished cells that were exclusively located in a single cross-sectional sector from those that wrapped around a larger fraction of the DA (see Fig S16).

We made the following changes to the supplementary material:

Page 17 of S1 Appendix: We added subsection 9.5 to S1 Appendix.

We added Fig S16.

Reviewer #2:

The paper presents a novel mathematical morphology model to study dorsal-ventral asymmetry in the morphology of endothelial cells within the dorsal aorta of zebrafish embryos, both wild-type and those deficient in Endoglin. Endothelial cells, lining blood vessel lumens, undergo shape changes in response to altered blood flow which affecting vessel geometry by elongating in the flow direction and altering the vessel's diameter. These changes are critical during early embryonic development, particularly between 48 and 72 hours post-fertilization, and require Endoglin—a protein whose absence leads to excessive endothelial cell growth and increased vessel diameter. This manuscript is generally well describe the mathematical model analyzing dorsal-ventral asymmetry of endothelial cell morphology in the dorsal aorta of zebrafish embryos introduces a novel and sophisticated approach to understanding complex biological structures.

We appreciate the positive feedback on the manuscript.

Here are some points that could be considered to improve the manuscript.

The model's robustness against variations in parameter values should be a standard and clearly defined. For example, why and where the parameters are from.

We thank the reviewer for making us aware of missing details regarding the choice of our tuning parameters. We would like to clarify that our novel mathematical approach is fine-tuned by 13 carefully designed algorithmic parameters (see also Table S2). Within the manuscript, we introduced each of these parameters within the respective algorithmic step within the Materials and methods section. An example for this can be seen in lines 213–216 of the clean manuscript:

"To ensure that the point density on all edges was comparable, we scaled the number $n_{\text{interp}}(p)$ of points interpolated on contour edge (p, p^+) with the edge length:

$$n_{\text{interp}}(p) = \left\lfloor n_{\text{interp,rel}} \cdot \left\| p^+ - p \right\|_2 \right\rfloor,$$

where $n_{\text{interp,rel}}$ is a tuning parameter controlling the relative number of points interpolated per unit length (here: µm) on edges of any contour."

After all tuning parameters had been introduced, we detailed the combined choice of these tuning parameter values in the Materials and methods section 'Criteria for choice of tuning parameters'. We now clarified in the manuscript that we first introduce each tuning parameter and then describe their combined choice later in the text.

We would like to explain that our tuning parameters can be broadly categorized as (1) controlling the overall *precision* of cell contour description or vessel surface reconstruction, (2) capturing the *complexity of the underlying experimental data* and (3) directly controlling the *local quality of estimated* vessel cross-sections. *Precision* parameters were designed in such a way that contours and vessel cross-sections of different sizes can be analyzed and compared using the same parameter values. For our morphometric analysis, we chose each of these values sufficiently high (in case of $n_{\text{interp,rel}}$, $n_{\text{spline,rel}}$, M_{rel}, n_{poly} and $n_{\text{cross,rel}}$) or sufficiently low (in case of ϵ_{spline}) to ensure precise description of endothelial cell (EC) contours and vessel surfaces. Please note that most of these parameters naturally occur when discretizing parametric curves or tubular surfaces; the error threshold ϵ_{spline} is directly adapted from the article [3]. The *data-complexity* parameters r and Δh stem from our custom cell contour enrichment algorithm (see Fig 2); we identified suitable values for them by visual inspection.

The *local-estimation-quality* parameters σ, Z_σ and Z_ω are standard parameters of Gaussian weights or filters. Instead of using a fixed standard deviation for the truncated Gaussian weights during cross-section estimation, we instead designed a custom *local-estimation-quality* parameter n_{oct} that locally varies the Gaussian function's width to

account for varying cell contour point density along the vessel axis (see Fig 4). To aid in local unidentifiability problems (see Fig S10D), we introduced the *local-estimation-quality* parameter λ that controls the deviation of local cross-sectional shapes from the mean shape. Instead of employing standard L_1 or L_2 type constraints on the local cross-sectional parameters, we constrained the overall shape of the local cross-section. This increases interpretability of this constraint and further avoids problems with the different parameters' scales and units.

We chose default values for Z_ω and Z_σ but considered n_{oct}, λ and σ to be critical as they control trade-offs between the locality of estimated cross-sectional shapes, the physiological plausibility of these shapes and the overall smoothness of the vessel surface (see Fig S10). For this reason, following the parameters' choice, we analyzed the sensitivity of both vessel geometry and EC morphology with respect to these parameters and found that the chosen parameter values were indeed robust (see Table S3 and Table S4).

We have added clarifications to the manuscript describing the parameters' nature and the reasoning behind our sensitivity analysis.

We made the following changes to the Material and methods section:

Lines 164–167 of the clean manuscript:
Following description of each computational step with the introduction of all involved tuning parameters, we explain how we evaluated the goodness of fit of our approach, how we chose suitable tuning parameter values and, finally, how we validated our novel mathematical approach.

Lines 227–230 of the clean manuscript:
These curves minimize discontinuities in the third derivative while keeping the sum of squared distances between the points of the enriched contours and their projections onto the splines below or equal to a user-specified threshold $\epsilon_{spline,i} > 0$ *(corresponding to* S *in [3]).*

Lines 466–470 of the clean manuscript:
Our novel mathematical approach is fine-tuned by 13 parameters (see also Table S2). These tuning parameters can be broadly categorized as (1) controlling the overall precision of cell contour description or vessel surface reconstruction, (2) capturing the complexity of the underlying experimental data and (3) directly controlling the local quality of estimated vessel cross-sections.

Lines 473–478 of the clean manuscript:
In short, tuning parameters controlling contour preprocessing were identified by visual inspection. Suitable values for the data-complexity parameters r *and* Δh*, which control EC contour enrichment by neighbors, ensure that the combined information of neighboring cells on a cell-cell contact is integrated into each of the cells' contours. Their magnitude depends only on the cell contours' complexity, i.e., the cells' deviation from an ellipse/hexagon due to irregular boundaries and protrusions.*

Line 479 of the clean manuscript:
precision parameters $n_{interp,rel}$*,* ϵ_{spline} *and* $n_{spline,rel}$

Lines 483–484 of the clean manuscript:
precision parameters M_{rel}*,* n_{poly} *and* $n_{cross,rel}$

Line 486 of the clean manuscript:
Local-estimation-quality parameters Z_ω *and* Z_σ

Lines 490–491 of the clean manuscript:
local-estimation-quality parameters n_{oct}*,* λ *and* σ

Lines 500–502 of the clean manuscript:
As the shapes of estimated vessel cross-sections are directly controlled by the local-estimation-quality parameters n_{oct}*,* λ *and* σ*, we next tested whether the parameters' values were robust by performing a sensitivity analysis:*

We made the following change to the supplementary material:

Pages 17–19 of S1 Appendix: We updated Section 10 (analogous to the changes of the manuscript above).

Also, while the paper might present a novel approach, the real test of any model validity is important to accurately describe experimental observations. Questions arises regarding the comprehensiveness of the data used for validation

We would like to thank the reviewer for emphasizing the importance of model validation. We wish to highlight that, prior to quantification of cell morphology and vessel geometry, we first validated our novel mathematical approach by confirming that vessel cross-sections solely estimated from cell contours complied with vessel cross-sections that were manually annotated on an angiography signal (see Fig S11 and Table S5).

Experimental data usable for validation of EC surface morphology measurements are generally scarce. This is due to the limited availability of mathematical approaches that can compute cell *surface* morphology from 3D microscopy images. The article by Sugden et al. [4] belongs to a few studies where both EC surface morphology and vessel geometry were quantified. This was possible by combining mathematical unrolling of EC contours and manual vessel diameter measurements. In that study, the authors quantified drastic changes in both EC morphology and vessel geometry in wild-type and Endoglin-deficient zebrafish DAs between 48 hpf and 72 hpf. Thus, we deemed these phenotypes and developmental conditions as a suitable system to evaluate the accuracy of our novel mathematical approach in computing EC morphology and vessel geometry.

To our knowledge, no other articles have been published that quantify EC morphology in the DA of Endoglin-deficient embryos. Even in wild-type embryos, cell surface morphology, like surface area, is rarely quantified in the DA [2, 5, 6]. We however identified additional articles (manually) measuring DA diameters [7, 1, 8, 9, 10, 6, 11, 12, 13]. To enable the reader to better judge our results in comparison to existing literature, we have expanded Tables S6 and S7 with data from the aforementioned references and also adapted the discussion accordingly. For details, see below.

We made the following change to the discussion:

Lines 850–903 of the clean manuscript:
As an additional evaluation of the quality of our experimental data and the accuracy of our cell contour-based vessel surface reconstruction, we scanned the literature for data on DA geometry and morphology of its constituent ECs. While we found many studies measuring DA geometry in wild-type embryos [1, 8, 7, 4, 5, 9, 10, 11, 6, 13, 12], we only identified two studies reporting DA diameters in Endoglin-deficient embryos [4, 11]. Overall, our measurements of DA geometry are in line with the high variability found in the literature (see Table S6): The majority of the reported means/medians of luminal area [5] and luminal diameter [7, 4, 10, 11, 13, 12] maximally differ by 20 % from our measurements. Also, the single observations of wild-type DA diameters

reported in [1] are within the range of our measurements. Notably smaller diameters in wild-type DAs at 48 hpf *and* 72 hpf *were reported in [7, 10, 6] and [9], respectively. The authors in [8] measured drastically bigger diameters in wild-types at* 72 hpf*.*

We consider two potential explanations for the observed differences in DA diameter: Firstly, vessel diameters are typically measured in 2D slices or projections of 3D images. In these planar views, it is difficult to ensure that diameter measurements are perpendicular to the vessel's anterior-posterior (and left-right) axis. But this is necessary as the greatest luminal extension is typically found in dorsal-ventral direction. Secondly, differences between the developmental stages of the analyzed zebrafish embryos can impact geometric measurements: In any study, the process of imaging an entire group of zebrafish embryos takes several hours. These delays during image acquisition increase between-embryo variability. This is especially critical as drastic changes in DA diameter occur during the short time interval analyzed here [4].

Data on EC surface morphology in the DAs of wild-type and Endoglin-deficient embryos are scarce (see Table S7). This is due to the necessity of mathematical approaches to accurately compute morphometric measures like cell surface area. In [4], unrolling of single EC contours allowed the authors to quantify 2D EC morphology in DAs of wild-type and Endoglin-deficient embryos at 48 hpf *and* 72 hpf*. While the majority of their mean surface areas and perimeters maximally differ by* 20 % *from our mean measurements, they reported (*30 %–31 %*) larger mean surface areas at* 72 hpf*. Their greater surface areas at this time point could potentially be explained by differences between the developmental stages of the analyzed embryos (discussed above), differences in the experimental setup or uncertainty during manual annotation.*

Computing unrolled maps of wild-type DAs allowed the authors in [2] to quantify 2D EC morphology at 48 hpf*. Their (*46 %*) larger mean EC surface area can partly be explained by the authors' separation of ECs and potentially hemogenic cells based on the cells' morphology and position within the DA; mean area of the latter cells differed less than* 20 % *from our ECs. Additionally, their 2D representation of the DA cannot accurately account for radial and translational variation of vessel geometry. The hereby resulting distortion of 2D contours might have affected computations of surface area.*

Although the authors in [5] did not leverage information on EC contours in their approach, they were able to compute averaged EC surface areas from the DA's surface area and the number of EC nuclei within the DA. While many of their mean surface areas in wild-types are in line with ours, they repeatedly measured (more than 20 %*) smaller mean surface areas at* 48 hpf*. Our larger mean EC sizes at this time point could be explained by our decision to exclude small round ventral cells during annotation. As these cells are likely to be extruding from the DA, they do not contribute to vessel geometry over a substantial time span.*

Finally, the authors in [6] measured drastically (at least 67 %*) lower mean EC surface areas in wild-type zebrafish at* 48 hpf *than we did. These large differences can be explained by the authors' approximation of EC surface area based on 2D microscopy slices. Analyzing EC surface area in a planar view neglects the cells' curvature and thus results in smaller area measurements. To limit errors made using this approximation, the authors excluded all ECs that wrapped out of view of the analyzed slice; these (large) cells however strongly impact mean area measurements.*

We made the following changes to the supplementary material:

We expanded Table S6 and Table S7.

and whether the model can generalize well.

We thank the reviewer for raising this point. To allow generalization and application of our mathematical approach to other experimental data, we designed it in a modular manner. Within the article, we already demonstrated that our approach is readily applicable to different developmental stages (48 hpf and 72 hpf) of the dorsal aorta (DA) in multiple phenotypes (wild-type and Endoglin-deficient embryos). Application of our approach to vessels with similar shapes but different sizes is made possible by fine-tuning our approach's parameter values (see also our response to the reviewer's question above).

During model validation, we showcased that our approach is able to employ different cross-sectional shapes like circles and ellipses (see Fig S11) and can thus be tailored to vessels with various geometry. The python implementation of our approach additionally allows for simple addition of further cross-sectional shapes due to its object-oriented nature. While we leveraged the superelliptic shape model's asymmetry during the coordinate system's estimation, it is alternatively also possible to incorporate further data to allow identification of a physiologically plausible coordinate system when employing other cross-sectional shapes. For example, information from neighboring vessels can be leveraged to determine a vessel's dorsal-ventral axis as done in [14].

Finally, we expect our model to also be generalizable to non-straight blood vessels by replacing the straight anterior-posterior axis by a curve. Vessel cross-sections would then be located on planes locally perpendicular to this curve as in [15].

Also, is there any way that the mathematical model can show how endoglin expression affects the morphology of cells or vessel geometry? such as chemical reactions.

The research results presented in the present article are part of a larger project in which we aim to better understand vascular remodeling by using a combination of mathematical modeling techniques from geometry, biochemical reaction kinetics and mechanics. Within this larger context, we consider vascular remodeling a multiscale process in which changes in blood flow dynamics, especially wall shear stress, affect signaling pathways in single ECs. Altered signaling dynamics on the EC level result in morphological changes, e.g., elongation of these cells in the direction of blood flow. Morphological changes on the single EC level can then translate into changes in blood vessel geometry, e.g., decreases in vessel diameter. Since alterations in vessel geometry change blood flow parameters like wall shear stress again, vascular remodeling is a dynamic feedback cycle.

A mathematical multiscale model that incorporates all the aforementioned processes would enable to analyze the effect of individual proteins like Endoglin on changes in EC morphology and vessel geometry. Due to the complexity of the multiscale feedback cycle, the full development of this model is out of the scope of the submitted article. Nevertheless, by enabling precise quantification and explanation of cell morphology and vessel geometry at fixed time points, the submitted approach is a necessary first step towards the development of such a dynamic model.

Reviewer #3:

The authors present a new methodological pipeline that allows for three-dimensional reconstructions of vessel lumens based off the contours of individual endothelial cells. Their method focuses on accurately characterizing the morphology of each individual endothelial cell within the vessel wall environment. The single cell distinction allows the authors to characterize the dynamics of different groups of endothelial cells – ventral vs dorsal vs left/right, as well as consider adjacent lumen vessel cross-sectional area.

> We appreciate the accurate summary of our manuscript.

While the authors methods may fall under a mathematical model in the sense that they capture essential features of a biological system in mathematical terms, the title and framing of their methodological pipeline as a simple 'mathematical model' seem misleading as it does not capture the fact that the user of said model must start from microscopy images, contour them and accurately reconstruct them in three-dimensions.

> We thank the reviewer for making us aware of the misleading usage of the term 'mathematical model'. To emphasize that the novel methodology described in this article requires careful manual cell contour annotation and involves several algorithmic steps that require parameter tuning and visual inspection of intermediate results, we replaced the term 'mathematical model' by 'mathematical approach' throughout the article. For an explanation of the additional changes made to the manuscript's title, see our response to the reviewer's comment directly below.
>
> We made the following change to the manuscript title:
>
> *Novel mathematical approach to accurately quantify 3D endothelial cell morphology and vessel geometry based on fluorescently marked endothelial cell contours: Application to the dorsal aorta of wild-type and Endoglin-deficient zebrafish embryos*
>
> Throughout the article, we changed the term 'mathematical model' to 'mathematical approach'. For example, we made the following change to the abstract:
>
> *To understand how these changes in vessel geometry emerge from morphological changes of individual endothelial cells, we developed a novel mathematical approach that allows 3D reconstruction and quantification of both dorsal aorta geometry and endothelial cell surface morphology.*

Overall, the approach of the manuscript is not hypothesis driven but rather technological, outlining a carefully laid out methodology for measuring key features of dorsal aorta anatomy from the single cell to vessel level that is applied to two organism models (control and mutant zebrafish models) at two different time points. Even though a mutant model is used the paper doesn't read as if the system was challenged in order to understand underlying biological mechanisms. It reads as a descriptive validation of the methodology. The impact of the paper and its accessibility can be improved by complete re-organization of the figures and more focusing of the text.

> We highly appreciate the reviewer's constructive feedback aiming at increasing the impact of the present work. We would like to clarify that the progress in the research of vascular remodeling is currently limited by the low number of available approaches that enable detailed quantification of both vessel geometry and endothelial cell (EC) morphology in 3D. For this reason, we aimed to provide a novel mathematical approach that is capable of simultaneously and consistently describing and quantifying cross-sectional geometry and endothelial cell surface morphology.

Before employing our novel tool in hypothesis-driven research, we ensured to thoroughly validate it within this article (see Fig S11 and Table S5). We further illustrated our approach's potential to generate novel biological insights by identification of a previously unrecognized dorsal-ventral asymmetry of EC morphology in the dorsal aorta (DA) of wild-type and Endoglin-deficient zebrafish embryos (see Fig 11). Taken together our manuscript should enable its readers to judge our approach's validity and usefulness when applying it to their own data.

We would like to emphasize that we understand the biological aspects of the reviewer. From our own experience, however, we know that writing an article that contains the description and validation of a novel methodology while also answering a biological question cannot do justice to both, or would end up being excessively long.

To emphasize that our article's focus is on the description and validation of a novel methodology, we made the following change to the manuscript's title:

Novel mathematical approach to accurately quantify 3D endothelial cell morphology and vessel geometry based on fluorescently marked endothelial cell contours: Application to the dorsal aorta of wild-type and Endoglin-deficient zebrafish embryos

Major points:

+ Apart from a small portion of the methodology overview (Fig. 1) there are no in-vivo images of the dorsal aorta or endothelial cells. Even in Figure 1, the individual cells cannot be distinguished. More importantly, it appears that the reader would have to download the whole package suite/all results in order to see a manual outline of an endothelial cell. All cell morphology images are cartoon schematics. Please include at least one annotation image of the cell data within the paper. Would be great to see cell contour enrichment on top of an actual cell for example.

We thank the reviewer for making us aware of insufficient illustrations of intermediate results in our article. To better understand our manual annotation process, we added a supplementary figure that shows how we annotated cell contours, coordinate system axes and angiogram slices in Imaris software (see Fig S7). Additionally, we included two supplementary figures showcasing (i) the change of cell contours after each computational step of our mathematical approach (see Fig S8), and (ii) the difference between the estimated coordinate system from the original coordinate system within the mounting medium (see Fig S9). Throughout the text, we added references to these illustrations.

Finally, to allow the reader of our article to better evaluate the accuracy of our manual annotation, we uploaded the Imaris files containing both the 3D confocal microscopy images of the DA and the manual annotations to a second Zenodo repository. To simplify comparison of our algorithm's output to the original biological images, we also added processed EC contours, i.e., the results of enrichment, smoothing and projection, to these files.

Of note, we would like to clarify that the contours and surfaces shown in the middle three rows of Fig 1 are actual (intermediate) results derived from contours that we manually annotated on the microscopy image shown in the top row of Fig 1. We changed the figure's caption to emphasize that the shown contours are not cartoons but actual (intermediate) results.

We made the following changes to the Materials and methods section:

Lines 172–174 of the clean manuscript:
Within a 3D reconstruction of the fluorescent signal from confocal z-stacks, we manually outlined Pecam1-EGFP-marked cell junctions using the "Measurements" tool (see Fig S7A).

Lines 187–189 of the clean manuscript:
Additionally, we annotated vectors, defined as two ordered points, that provided approximate directions of anterior-posterior, dorsal-ventral and left-right axes (see Fig S7B).

Lines 194–196 of the clean manuscript:
Using the "Ortho slicer" tool, we manually annotated the outlines of several cross-sections along the anterior-posterior axis of the DA with the "Measurements" tool (see Fig S7C).

Lines 219–220 of the clean manuscript:
To see an exemplary outcome of cell contour enrichment, compare panels A and B of Fig S8.

Lines 246–247 of the clean manuscript:
To see an exemplary outcome of cell contour smoothing, compare panels B and C of Fig S8.

Lines 267–269 of the clean manuscript:
The difference between the coordinate system given by the embryo's position within the mounting medium and the estimated coordinate system are exemplified in Fig S9.

Lines 347–348 of the clean manuscript:
To see an exemplary outcome of vessel surface smoothing, compare panels (iii) and (iv) of Fig S10E.

Lines 378–379 of the clean manuscript:
To see an exemplary outcome of cell contour projection, compare panels C and D of Fig S8.

Lines 547–553 of the clean manuscript:
The analysis' underlying microscopy images including the manually annotated EC contours are available from the Zenodo database via https://doi.org/10.5281/zenodo.11353016. These files can be inspected using the free Imaris Viewer software (Bitplane). To allow comparison of the manually annotated EC contours against their respective enriched contours, smoothing splines and their projections onto the estimated vessel surfaces, we additionally inserted these intermediate results into the Imaris files.

We made the following change to the caption of Fig 1:

The 3D microscopy image in the top row stems from Endoglin-deficient embryo 13 at 72 hpf *and was used for the subsequent computations in the rows below.*

We made the following changes to the supplementary material:

We added Fig S7, Fig S8 and Fig S9.

+ The authors frequently refer to 'good agreement with results reported in [ref]' without acknowledging why ref was so important (was it the only instance that anyone has measured said value, etc) or what method was used in the ref. (Example page 5 line 87; page 33 line 798, 804, 806, 807).

We thank the reviewer for making us aware of missing explanations within the manuscript. Experimental data usable for validation of endothelial cell surface morphology measurements are generally scarce. This is due to the limited availability of mathematical approaches that can compute cell *surface* morphology from 3D microscopy images. The article by Sugden et al. [4] belongs to a few studies where both EC surface morphology and vessel geometry were quantified. This was possible by combining mathematical unrolling of EC contours and manual vessel diameter measurements. In that study, the authors quantified drastic changes in both EC morphology and vessel geometry in wild-type and Endoglin-deficient zebrafish DAs between 48 hpf and 72 hpf. Thus, we deemed these phenotypes and developmental conditions as a suitable system to evaluate the accuracy of our novel mathematical approach in computing EC morphology and vessel geometry.

To our knowledge, no other articles have been published that quantify EC morphology in the DA of Endoglin-deficient embryos. Even in wild-type embryos, cell surface morphology, like surface area, is rarely quantified in the DA [2, 5, 6]. We however identified additional articles (manually) measuring DA diameters [7, 1, 8, 9, 10, 6, 11, 12, 13]. To enable the reader to better judge our results in comparison to existing literature, we have expanded Tables S6 and S7 with data from the aforementioned references and also adapted the discussion accordingly. For details, see below.

We made the following change to the discussion:

Lines 850–903 of the clean manuscript:
As an additional evaluation of the quality of our experimental data and the accuracy of our cell contour-based vessel surface reconstruction, we scanned the literature for data on DA geometry and morphology of its constituent ECs. While we found many studies measuring DA geometry in wild-type embryos [1, 8, 7, 4, 5, 9, 10, 11, 6, 13, 12], we only identified two studies reporting DA diameters in Endoglin-deficient embryos [4, 11]. Overall, our measurements of DA geometry are in line with the high variability found in the literature (see Table S6): The majority of the reported means/medians of luminal area [5] and luminal diameter [7, 4, 10, 11, 13, 12] maximally differ by 20 % *from our measurements. Also, the single observations of wild-type DA diameters reported in [1] are within the range of our measurements. Notably smaller diameters in wild-type DAs at* 48 hpf *and* 72 hpf *were reported in [7, 10, 6] and [9], respectively. The authors in [8] measured drastically bigger diameters in wild-types at* 72 hpf.

We consider two potential explanations for the observed differences in DA diameter: Firstly, vessel diameters are typically measured in 2D slices or projections of 3D images. In these planar views, it is difficult to ensure that diameter measurements are perpendicular to the vessel's anterior-posterior (and left-right) axis. But this is necessary as the greatest luminal extension is typically found in dorsal-ventral direction. Secondly, differences between the developmental stages of the analyzed zebrafish embryos can impact geometric measurements: In any study, the process of imaging an entire group of zebrafish embryos takes several hours. These delays during image acquisition increase between-embryo variability. This is especially critical as drastic changes in DA diameter occur during the short time interval analyzed here [4].

Data on EC surface morphology in the DAs of wild-type and Endoglin-deficient embryos are scarce (see Table S7). This is due to the necessity of mathematical approaches to accurately compute morphometric measures like cell surface area. In [4], unrolling of single EC contours allowed the authors to quantify 2D EC morphology in DAs of wild-type and Endoglin-deficient embryos at 48 hpf *and* 72 hpf. *While the majority of their mean surface areas and perimeters maximally differ by* 20 % *from our mean measurements, they reported* (30 %–31 %) *larger mean surface areas at* 72 hpf. *Their*

greater surface areas at this time point could potentially be explained by differences between the developmental stages of the analyzed embryos (discussed above), differences in the experimental setup or uncertainty during manual annotation.

Computing unrolled maps of wild-type DAs allowed the authors in [2] to quantify 2D EC morphology at 48 hpf*. Their (*46 %*) larger mean EC surface area can partly be explained by the authors' separation of ECs and potentially hemogenic cells based on the cells' morphology and position within the DA; mean area of the latter cells differed less than* 20 % *from our ECs. Additionally, their 2D representation of the DA cannot accurately account for radial and translational variation of vessel geometry. The hereby resulting distortion of 2D contours might have affected computations of surface area.*

Although the authors in [5] did not leverage information on EC contours in their approach, they were able to compute averaged EC surface areas from the DA's surface area and the number of EC nuclei within the DA. While many of their mean surface areas in wild-types are in line with ours, they repeatedly measured (more than 20 %*) smaller mean surface areas at* 48 hpf*. Our larger mean EC sizes at this time point could be explained by our decision to exclude small round ventral cells during annotation. As these cells are likely to be extruding from the DA, they do not contribute to vessel geometry over a substantial time span.*

Finally, the authors in [6] measured drastically (at least 67 %*) lower mean EC surface areas in wild-type zebrafish at* 48 hpf *than we did. These large differences can be explained by the authors' approximation of EC surface area based on 2D microscopy slices. Analyzing EC surface area in a planar view neglects the cells' curvature and thus results in smaller area measurements. To limit errors made using this approximation, the authors excluded all ECs that wrapped out of view of the analyzed slice; these (large) cells however strongly impact mean area measurements.*

We made the following changes to the supplementary material:

We expanded Table S6 and Table S7.

+ The authors are commended for studying intra-embryo and between embryo variability. Given the complexity and length of the dorsal aorta it seems normal that relative position would play a role in variability, so perhaps it is not necessary to report detailed endothelial cell morphology variations without stratification.

We thank the reviewer for making us aware of an imprecise description of our methodology. To generate comparable data sets, we reduced within-embryo and between-embryo variability by annotating cell contours in the identical DA segment in each embryo. This segment of the DA is positioned posterior to the tip of the yolk extension and ranges over 3–4 intersegmental vessels. Thus, the observed high variability of EC morphology is not caused by differences in the cells' position along the DA's anterior-posterior axis. Instead, we demonstrated in Fig 11 that stratification of cells by their cross-sectional (and not their anterior-posterior) location within the DA further reduced the remaining high within-embryo variability in EC morphology. Our manuscript now includes a clearer description of the analyzed DA segment's position.

We made the following change to the Materials and methods section:

Lines 174–177 of the clean manuscript:
To reduce within-embryo and between-embryo variability, we annotated cell contours always within the same vessel segment in each embryo; the annotated segment is positioned posterior to the tip of the yolk extension and ranges over 3–4 intersegmental vessels.

+ so many figures, including the novel findings of dorsal-ventral asymmetry endothelial cell morphology are summarized in box plots (Fig. 11). Please couple these box plots with some images and schematics to really highlight the importance of this methodology and its applications. Page 29 lines 681 -694 are so interesting. It would be great to visualize the phenomenon within. Along these lines it would also be nice to see the 'dorsal aorta flattening' observed in 3D microscopy images.

> We thank the reviewer for the very positive feedback. Solely for the purpose of visualization of the dorsal-ventral asymmetry of EC morphology (including the findings of lines 681–694 of the old manuscript version), we added Fig S17. For this figure, we simplified blood vessel geometry: We described vessel cross-sections by the (constant) mean shape along the entire length of the analyzed segments, thus neglecting potentially important variations in local geometry. This allowed us to unroll the vessel surface in a second step circumventing cartographic distortions and illustrate the differential morphology of dorsal and ventral ECs in 2D. Note however that the hereby resulting increases in contour projection distance were substantial for some of the analyzed cells.
>
> To illustrate that cross-sections of the DA can be flattened *dorsally*, while still being round ventrally, we added supplementary Fig S1. Here, we took snapshots in the original 3D microscopy images of the validation data, i.e., we show the merged signal of the DA's Dextran-perfused lumen and the surrounding Pecam1-EGFP-labeled EC junctions.
>
> We made the following change to the introduction:
>
> Lines 69–72 of the clean manuscript:
> *While all the aforementioned approaches described DA cross-sections using circles or ellipses [4, 2, 14], we repeatedly observed in 3D microscopy images that dorsal segments of DA cross-sections can be flattened in comparison to their ventral segments (see Fig S1).*
>
> We made the following change to the Results section:
>
> Lines 793–799 of the clean manuscript:
> *Solely for the purpose of visualization of the identified morphological differences between dorsal and ventral ECs, we further simplified blood vessel geometry: We described vessel cross-sections by the (constant) mean shape along the entire length of the analyzed segments, thus neglecting potentially important variations in local geometry. This allowed us to unroll the vessel surface in a second step circumventing cartographic distortions (see Fig S17). Note however that the hereby resulting increases in contour projection distance were substantial for some of the analyzed cells.*
>
> We made the following changes to the supplementary material:
>
> Page 19 of S1 Appendix: We added Section 11.
>
> We added Fig S1 and Fig S17.

Other comments:
+page 3 lines 19-21 would be nice for the 'drastic changes' that the dorsal aorta undergoes from 48hpf to 72hpf to be captured in a schematic

> We thank the reviewer for this suggestion that allows better understanding of the biological processes that are central to our study. In their work, Sugden et al. already

illustrated this phenomenon well in their supplement [4]. We now explicitly refer to their supplementary figure in our introduction.

We made the following change to the introduction:

Lines 19–21 of the clean manuscript:
The dorsal aorta (DA) is the major artery in the zebrafish trunk and undergoes drastic remodeling during embryonic development (for an illustration, see Supplementary Figure 7 in [4]):

+ For the major challenge of vessel reconstructions with images lying on one side, is it not possible to flip the embryos within their eggs?

We thank the reviewer for making us aware of ambiguities regarding our image acquisition. We would like to better explain why embryos were imaged from one side: Embryos were dechorionated, so they were not anymore within 'their egg', rather the embryos were stretched out. Due to their flat morphology, the embryos naturally lay on either side. Imaging from the lateral side also provided best visibility (least tissue interference) for confocal microscopy.

Due to the bilateral symmetry of the DA and the surrounding tissue, information gained by imaging from either the left or right side is equivalent. In this study, we thus chose to place all embryos on their right side. After imaging the embryos from the right side, it would be possible to flip the embryos onto their left side and repeat the image acquisition process. While this would produce a second microscopy image with improved fluorescence signal quality of the left side, it would be hard to merge the image from the left with the image from the right into a single consistent image (also due to time delays). We now explain in our manuscript why we imaged embryos from the right side.

We made the following change to the Materials and methods section:

Lines 143–148 of the clean manuscript:
As dechorionation flattened the embryos' overall morphology, they naturally lay on either side in the microscope. Imaging them from the lateral side also ensured least tissue interference for confocal microscopy. Due to the bilateral symmetry of the DA and the surrounding tissue, information gained by imaging from either the left or right side is equivalent. In this study, we thus chose to place all embryos on their right side in the microscope.

References

[1] Kissa K, Herbomel P. Blood stem cells emerge from aortic endothelium by a novel type of cell transition. Nature. 2010;464(7285):112–115. doi:10.1038/nature08761.

[2] Lancino M, Majello S, Herbert S, De Chaumont F, Tinevez JY, Olivo-Marin JC, et al. Anisotropic organization of circumferential actomyosin characterizes hematopoietic stem cells emergence in the zebrafish. eLife. 2018;7. doi:10.7554/eLife.37355.

[3] Dierckx P. Algorithms for smoothing data with periodic and parametric splines. Computer Graphics and Image Processing. 1982;20(2):171–184. doi:10.1016/0146-664x(82)90144-7.

[4] Sugden WW, Meissner R, Aegerter-Wilmsen T, Tsaryk R, Leonard EV, Bussmann J, et al. Endoglin controls blood vessel diameter through endothelial cell shape changes in response to haemodynamic cues. Nature cell biology. 2017;19:653–665. doi:10.1038/ncb3528.

[5] Campinho P, Lamperti P, Boselli F, Vilfan A, Vermot J. Blood Flow Limits Endothelial Cell Extrusion in the Zebrafish Dorsal Aorta. Cell reports. 2020;31:107505. doi:10.1016/j.celrep.2020.03.069.

[6] Greysson-Wong J, Rode R, Ryu JR, Chan JL, Davari P, Rinker KD, et al. rasa1-related arteriovenous malformation is driven by aberrant venous signalling. Development (Cambridge, England). 2023;150. doi:10.1242/dev.201820.

[7] Jörgens K, Stoll SJ, Pohl J, Fleming TH, Sticht C, Nawroth PP, et al. High tissue glucose alters intersomitic blood vessels in zebrafish via methylglyoxal targeting the VEGF receptor signaling cascade. Diabetes. 2015;64:213–225. doi:10.2337/db14-0352.

[8] Zhao D, Qin C, Fan X, Li Y, Gu B. Inhibitory effects of quercetin on angiogenesis in larval zebrafish and human umbilical vein endothelial cells. European journal of pharmacology. 2014;723:360–367. doi:10.1016/j.ejphar.2013.10.069.

[9] Lu YA, Je JG, Hwang J, Jeon YJ, Ryu B. Ecklonia cava Extract and Its Derivative Dieckol Promote Vasodilation by Modulating Calcium Signaling and PI3K/AKT/eNOS Pathway in In Vitro and In Vivo Models. Biomedicines. 2021;9. doi:10.3390/biomedicines9040438.

[10] Maung Ye SS, Kim JK, Carretero NT, Phng LK. High-Throughput Imaging of Blood Flow Reveals Developmental Changes in Distribution Patterns of Hemodynamic Quantities in Developing Zebrafish. Frontiers in physiology. 2022;13:881929. doi:10.3389/fphys.2022.881929.

[11] Snodgrass RO, Govindpani K, Plant K, Kugler EC, Doh C, Dawson T, et al. Therapeutic targeting of vascular malformation in a zebrafish model of hereditary haemorrhagic telangiectasia. Disease models & mechanisms. 2023;16. doi:10.1242/dmm.049567.

[12] Hasan M, Zedan HT, Al-Fakhroo D, Elsayed Ibrahim H, Abiib SI, El-Sherbiny IM, et al. In vivo testing of novel nitric oxide-releasing nanoparticles for alleviating heart failure using the zebrafish embryo model. Nitric oxide: biology and chemistry. 2024;144:47–57. doi:10.1016/j.niox.2024.01.007.

[13] Schumacher JA, Wright ZA, Rufin Florat D, Anand SK, Dasyani M, Batta SPR, et al. SH2 domain protein E and ABL signaling regulate blood vessel size. PLoS genetics. 2024;20:e1010851. doi:10.1371/journal.pgen.1010851.

[14] Campinho P, Lamperti P, Boselli F, Vermot J. Three-dimensional microscopy and image analysis methodology for mapping and quantification of nuclear positions in tissues with approximate cylindrical geometry. Philosophical transactions of the Royal Society of London Series B, Biological sciences. 2018;373. doi:10.1098/rstb.2017.0332.

[15] Yang R, Li E, Kwon YJ, Mani M, Beitel GJ. QuBiT: a quantitative tool for analyzing epithelial tubes reveals unexpected patterns of organization in the ,

jakarta.xml.bind.JAXBElement@741e0987, trachea. Development (Cambridge, England). 2019;146. doi:10.1242/dev.172759.
